# Potential Application of Plant By-Products in Biomedicine: From Current Knowledge to Future Opportunities

**DOI:** 10.3390/antiox14080942

**Published:** 2025-07-31

**Authors:** Silvia Estarriaga-Navarro, Teresa Valls, Daniel Plano, Carmen Sanmartín, Nieves Goicoechea

**Affiliations:** 1 Department of Pharmaceutical Sciences, School of Pharmacy and Nutrition, University of Navarra, Irunlarrea 1, 31008 Pamplona, Spain; sestarriagan@unav.es (S.E.-N.); dplano@unav.es (D.P.); sanmartin@unav.es (C.S.); 2 Department of Environmental Biology-Plant Stress Physiology Group, Associated to CSIC (EEAD, Zaragoza, Spain), BIOMA Institute for Biodiversity and the Environment, University of Navarra, Irunlarrea 1, 31008 Pamplona, Spain; mvalls@alumni.unav.es; 3 Navarra Institute for Health Research (IdisNA), Irunlarrea 3, 31008 Pamplona, Spain

**Keywords:** antioxidant products, antimicrobial agents, antitumoral activity, circular economy, green education, healthcare industry 4.0, protocol standardization, vegetable by-products

## Abstract

Plant by-products have gained significant attention due to their rich content in bioactive compounds, which exhibit promising antioxidant, antimicrobial, and antitumor properties. In European countries, vegetable waste generation ranged from 35 to 78 kg per capita in 2022, highlighting both the scale of the challenge and the potential for valorization. This review provides an overview of key studies investigating the potential of plant residues in biomedicine, highlighting their possible contents of antioxidant compounds, their antimicrobial and antitumor properties, as well as their applications in dermocosmetics and nutraceuticals. However, despite their potential, several challenges must be addressed, such as the standardization of extraction protocols, as bioactive compound profiles can vary with plant source, processing conditions, and storage methods. Effective segregation and storage protocols for household organic waste also require optimization to ensure the quality and usability of plant by-products in biomedicine. Emerging 4.0 technologies could help to identify suitable plant by-products for biomedicine, streamlining their selection process for high-value applications. Additionally, the transition from in vitro studies to clinical trials is hindered by gaps in the understanding of Absorption, Distribution, Metabolism, and Excretion (ADME) properties, as well as interaction and toxicity profiles. Nonetheless, environmental education and societal participation are crucial to enabling circular bioeconomy strategies and sustainable biomedical innovation.

## 1. Introduction

Currently, the pharmaceutical sector is showing a growing interest in the use of vegetable residues, primarily due to their richness in high-value-added bioactive molecules. These plant-derived residues, often generated during fruit and vegetable processing, include by-products such as pomace, seeds, leaves, pulp, peels, and stems. Far from being mere waste, these materials are recognized for their substantial content of carbohydrates, proteins, fibers, polyphenols, and lipids, compounds that exhibit significant nutritional and pharmacological potential [1,2,3,4]. An increasing number of studies have highlighted the diverse biological activities of these compounds, including anti-inflammatory, antioxidant, antibacterial, antiparasitic, and antifungal effects, positioning them as promising candidates for the development of novel therapeutic products [5,6]. Furthermore, these by-products have been successfully applied in the cosmetic industry, contributing to the formulation of skincare products and treatments that harness their natural bioactive potential. Beyond their conventional applications, vegetable residues are increasingly being investigated for advanced biomedical uses, such as their incorporation into tissue engineering scaffolds and drug delivery systems [7,8]. These innovative applications are driving a paradigm shift in how agro-industrial waste is perceived, transforming it from an environmental burden into a valuable source of functional materials. Simultaneously, the food industry is investing in the development of emerging technologies aimed at valorizing plant by-products to create novel food products with enhanced nutritional profiles [9,10,11]. This trend not only supports the principles of the circular economy but also aligns with current consumer demands for sustainable and health-promoting products.

Plant residues are generated by the processing of horticultural crops, and later, by agri-food industries and in the domestic sphere. According to the estimations made by the United Nations, the world’s population will increase from 8 billion at the beginning of 2024 to 9.7 billion in 2050 [12], which represents a 21% increase. Consequently, the rising demand for food involves the production of increased amounts of waste and by-products in fulfilling the needs of the population.

**Figure 1:**
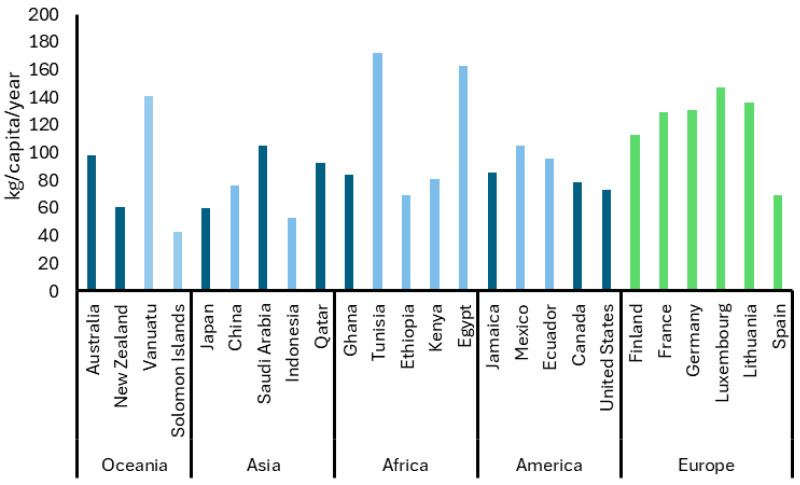
Global domestic food waste generation (kg/capita/year) by continent and country in 2022. The figure uses a color scheme that distinguishes the level of confidence associated with each data point. High confidence (dark blue) estimates are nationally representative and methodologically robust, making them reliable for tracking food waste. Medium confidence (light blue) estimates are less representative, often based on localized data or adjusted figures. Eurostat data (green) follows standardized methods across European countries and corresponds to data published in 2024 with updates in 2025 [13,14].

To provide insight into the scale of food waste generation, Figure 1 presents global data from 2022 on food waste per capita across different continents. It reveals significant disparities among continents. Countries such as Tunisia, Egypt, and Vanuatu report the highest values, though their estimates are based on medium-confidence data, which may limit the reliability of direct comparisons. In contrast, Australia, Saudi Arabia and Qatar show similarly high figures but with high confidence, suggesting stronger support for the reported values. European countries, represented with Eurostat data, show more moderate values clustered between 53 and 91 kg per capita, with relatively consistent patterns across the region [13]. Interestingly, although the United States, China, and Germany are the world’s three largest economies by Gross Domestic Product (GDP), their per-capita food waste levels do not show a consistent correlation with their economic performance. This suggests GDP alone does not explain food waste differences. Factors such as consumption habits, public policies, cultural values, and supply chain efficiency significantly influence food waste [14].

Building on this, it is also valuable to examine temporal trends within Europe to better understand how food waste is evolving in a high-income, highly regulated context. Between 2020 and 2022, per-capita food waste levels across European countries exhibited notable variation. For instance, Denmark and Cyprus showed marked increases of +33 kg and +21 kg per capita (2020–2022), respectively. In contrast, the Netherlands and Luxembourg experienced substantial reductions in the same period (–32 kg and –25 kg, respectively). Other countries such as Germany and Croatia maintained relatively stable figures, suggesting entrenched consumption and disposal patterns. Overall, the diversity of these trends highlights that even within a relatively homogeneous economic and regulatory area like the European Union (EU), national-level dynamics (including policy enforcement, public awareness campaigns, and food distribution systems) play a crucial role in shaping food waste outcomes [13].

**Figure 2:**
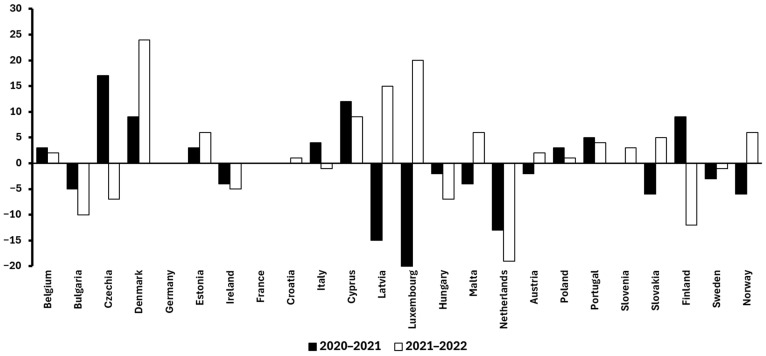
Variation in food waste generation (kg/capita) across European countries from 2020 to 2022 [13].

It is also important to consider differences in vegetable availability and accessibility between countries, as these factors can directly impact food waste levels (Figure 1 and Figure 2). For example, Spain’s strong local vegetable production, supported by favorable climate and shorter supply chains, likely contributes to more stable availability and accessibility, which may help reduce waste through fresher produce and better inventory management. In contrast, countries such as the Netherlands and Luxembourg depend heavily on imports, leading to greater seasonal fluctuations and supply chain challenges that can increase food waste due to spoilage or overstocking. These structural differences in vegetable supply chains are reflected in the varying food waste trends observed across Europe, as shown in the data: countries with more localized production tend to have smaller increases or even reductions in waste, whereas those reliant on imports may experience greater volatility in waste generation. Interestingly, Germany (despite relying heavily on imported vegetables) maintained stable food waste levels from 2020 to 2022. This relative stability can be attributed to effective infrastructure, efficient logistics, and the implementation of a robust national strategy [13].

**Figure 3:**
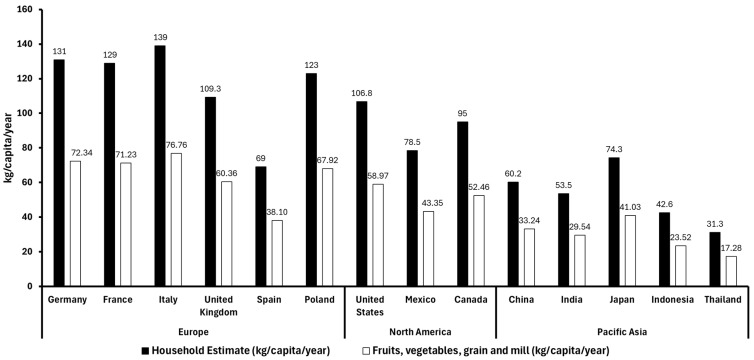
Total food waste (kg/capita/year) (black bars) and fruit, vegetable, grain, and cereal residues (kg/capita/year) (white bars) in different countries of Europe, America, and Asia in 2022 [13,16].

According to a pooled analysis conducted between 2010 and 2022, 55.22% of food waste in high-income countries is attributed to fruits, vegetables, and grain and mill products. Therefore, based on the database of global food waste statistics (Figure 1) [13,14], we have applied this percentage to estimate the proportion of food waste from fruits, vegetables, and grain and mill products in the Asia-Pacific, Europe, and North American regions (Figure 3). This suggests that plant-based waste contributes significantly to household food waste globally, with higher per-capita waste observed in countries with both strong local production and imports. For example, European countries like Italy and France, which produce large quantities of fruit, show high fruit waste per capita, while countries like Germany, relying more on imports, report lower fruit waste levels. In Asia, Indonesia and Thailand report lower fruit and vegetable waste levels mainly because of factors like lower overall consumption, more careful food use driven by economic constraints, and possibly less food surplus. Additionally, their food supply chains may be shorter or more localized in some areas, reducing spoilage before consumption. Cultural habits around minimizing waste and less access to overabundance in markets can also contribute to lower waste despite less production and reliance on imports [15].

In this context, the idea of the circular economy arose with the aim of replacing “take–make–dispose” practices. The circular model is based on substituting constant production with a focus on achieving sufficiency, maximizing reusability, recycling what cannot be repurposed, fixing what is damaged, and remanufacturing what cannot be broken [17]. The circular economy minimizes the use of natural resource inputs and the generation of waste, contaminants, and carbon emissions [9,18,19]. In addition, Regulation (EC) 2008/98 on waste establishes the legal framework for waste management and promotes waste prevention, reuse, and recycling, encouraging the valorization of food by-products as raw materials rather than waste [20]. The directive distinguishes between food waste and by-products. The first one refers to edible or inedible residual materials that are no longer suitable for consumption or use and are typically discarded without further valorization. This includes leftovers, spoiled food, or unused ingredients from the supply chain. In contrast, by-products are secondary materials generated unintentionally during food processing or manufacturing. Although they are not primary products, they retain nutritional, functional, or economic value and can be repurposed for various applications [21,22]. Within this framework, the valorization of vegetable by-products aligns perfectly with the core principles of the circular economy, as it promotes the transformation of the vast amounts of food by-products generated worldwide, which were traditionally regarded as worthless, into valuable raw materials. This approach not only reduces the environmental impact but also adds economic value to the substantial volumes of by-products generated. Agro-industrial by-products such as fruit peels, seeds, stems, and pomace are now being re-evaluated as renewable sources of bioactive compounds with broad applicability. Plant by-products are being studied for their antimicrobial, anti-inflammatory, and antioxidant properties in pharmaceuticals [23]. In biomedicine, they help develop sustainable materials like scaffolds and drug delivery systems [24]. The cosmetic industry uses them for skincare products, while in nutrition, these compounds contribute to health-promoting functional foods and supplements [25]. This valorization strategy supports a shift towards more sustainable production systems, where environmental impact is minimized and material loops are closed. It also opens up new avenues for innovation, encouraging cross-sector collaboration between agriculture, food processing, biotechnology, and health sciences.

**Figure 4:**
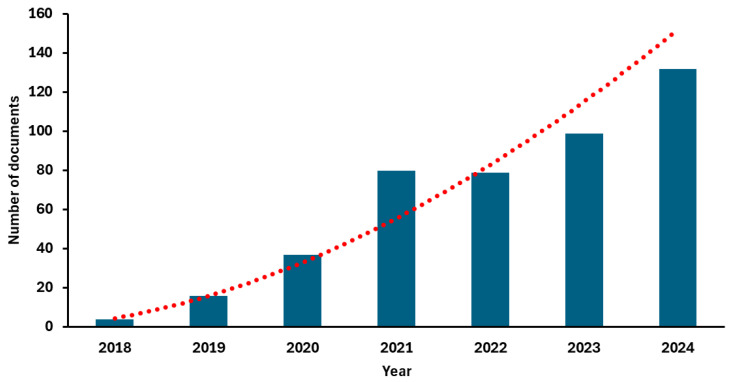
Number of documents focused on food by-products published from 2018 to 2024 according to data collected from PubMed using “food by-products and circular economy” as keywords [26]. The red line represents the fitted power trendline, illustrating the accelerating growth in the number of publications over time.

Accordingly, in recent years, this model has triggered an exponential growth in scientific research. As illustrated in Figure 4, the number of articles indexed in PubMed under the search terms “food by-products and circular economy” has increased significantly, reflecting the rising academic interest in this field [26].

With the goal of promoting research projects focused on the re-use of plant by-products, as well as the encouraging a circular economy, this review offers a comprehensive look at the potential health-related applications of these materials. The compiled information has allowed for performing a SWOT analysis (Strengths, Weaknesses, Opportunities, and Threats) focused on both the current situation and future perspectives on the application of agri-food plant by-products in biomedicine.

## 2. Materials and Methods

A systematic literature review was conducted using three major scientific databases: PubMed, Scopus, and Google Scholar, focusing on peer-reviewed articles published between 2013 and 2025. It was complemented by a search in the LENS patent database to capture innovations and intellectual property trends between 2013 and 2025.

To ensure broad and relevant coverage, a comprehensive set of keywords was used, including plant residues, plant by-products, circular economy, bioactive compounds, biomedical applications, antimicrobial, antifungal, antitumoral, dermocosmetics, biomaterials, prebiotics, nutraceuticals, phytochemicals, and green chemistry. These were supplemented with secondary terms such as sustainability, legislation, regulatory frameworks, economic feasibility, barriers, opportunities, innovation, green extraction methods, biorefineries, integral biorefineries, citizens education, and future perspectives.

Articles were selected following a two-stage screening process, beginning with title and abstract review, followed by full-text evaluation to ensure alignment with the scope of this review. Selection criteria emphasized scientific rigor, originality, and relevance to the valorization of plant residues in biomedical contexts. Only studies that clearly addressed the use or transformation of plant-derived by-products, such as peels, leaves, or stems, into bioactive compounds or functional materials for applications in health, medicine, or dermocosmetics were included. In particular, preference was given to experimental research (in vitro, in vivo, or clinical trials), systematic reviews, and meta-analyses that provided evidence-based insights into the therapeutic potential, mechanisms of action, or formulation strategies of these bioresources. Additionally, studies addressing broader dimensions such as technological challenges, regulatory frameworks, legislative developments, economic feasibility, and future implementation perspectives within the circular economy context were also prioritized, as they contribute to a more comprehensive understanding of the opportunities and limitations associated with the biomedical valorization of plant residues.

Research that did not specifically address the valorization of plant residues in biomedicine was excluded. Studies conducted in contexts outside biomedicine, such as those focusing solely on agriculture, were also excluded. The search was restricted to papers written in the English language.

## 3. Development Drivers of the Field

In recent years, there has been increasing interest in the efficient use of agricultural and food by-products as a means of improving sustainability, minimizing waste, and creating value-added products, which has stimulated scientific research (Figure 4) and technological advances [27]. In fact, there are several critical factors that have driven growth in the use of plant by-products, including increasing sustainability awareness, advances in material processing, and regulatory support.

Growing awareness of environmental sustainability is driving change across society and industries, encouraging practices that prioritize conserving resources. Sustainability aims to ensure that future generations have access to resources at least as abundant as those available today, preventing the depletion of natural resources. Current overconsumption represents an injustice to future generations, with negative impacts on the environment, economy, and society. Food waste, which leads to economic losses and worsens food insecurity, is a global issue with severe environmental consequences, such as greenhouse gas emissions [28]. In recent years, environmental sustainability awareness has grown significantly, with both young people and adults expressing deep concern over the planet’s future, especially in the context of climate change and environmental disasters [29]. In this context, there is an ever-increasing demand for products with highly valued properties (nutritional, healthcare, and cosmetic). Thus, fruit and vegetable by-products offer significant potential due to their abundance and their high levels of bioactive compounds [30].

The advancement in material processing technologies, including green emerging technologies for the extraction of bioactives (their strengths, limitations, and applications) and the development of integrated biorefineries, are detailed in Table S1 and Section 5. Conventional methods of extracting bioactive compounds from plant tissues include Soxhlet extraction, maceration, percolation, and hydro-distillation. Non-conventional, green, or emerging extraction methods include ultrasound-assisted extraction (UAE), microwave-assisted extraction (MAE), enzyme-assisted extraction (EAE), fermentation-assisted extraction (FAE), supercritical fluid extraction (SFE), pressurized liquid extraction (PLE), pulse electric field-assisted extraction (PEFAE), supercritical water extraction (SWE), instant controlled pressure drop (détente instantanée contrôllée, DIC), supercritical liquid chromatography (SFC), pressurized hot water extraction (PHWE), and deep eutectic solvents (DESs). The main advantages of these methods over conventional ones are shorter extraction times, reduced solvent usage, higher yields, high-quality extracts, greater selectivity, better isolation, reduced environmental impact, lower energy consumption, and safer products [31,32,33].

Economic and political support has further advanced the circular economy, with public procurement driving innovation in resource conservation. By creating demand and offering incentives for research and development, it encourages companies to adopt more sustainable practices. Additionally, it fosters collaboration between suppliers and provides political support, further accelerating the transition to more sustainable and circular systems [34]. Programs within the European Union, such as Horizon Europe, Next-Generation EU, and LIFE, provide significant funding to support circular economy initiatives [35]. Globally, there are frameworks like the “2030 Agenda for Sustainable Development” adopted by the United Nations in 2015, which outlines 17 Sustainable Development Goals (SDGs), including goals focused on sustainable consumption, climate change, and environmental protection (SDGs 12, 13, 14, and 15) [36,37,38]. As circular economy practices expand, regulatory frameworks are essential to ensure safe and appropriate reuse of by-products. Previous studies questioned the safety of valorized food by-products due to limited research on their potential risks. Concerns arose because many studies focused only on their beneficial properties, without adequate safety evaluations, such as microbiological tests or contaminants analysis [39]. An example of this issue occurred in Italy in 2019 [40], where the improper use of olive pomace led to a legal case. The company failed to demonstrate its correct use as a fertilizer according to the requirements, highlighting the need for clear regulations to ensure the safe and proper use of food by-products.

Nowadays, in the European Union, food by-products are required to comply with strict safety regulations to ensure food safety and consumer protection. These include Regulation (EC) 178/2002, which sets the principles for food law; regulation (EC) 396/2005, which sets maximum pesticide residue levels in food and feed; or regulation (EC) 2073/2005, which defines microbiological criteria for food safety. These regulations ensure that foods, including vegetable by-products, comply with European food safety standards and are safe for consumer use [41]. Furthermore, in parallel, increasing Food and Drug Administration (FDA) approvals of natural products, driven by toxicity studies, highlight the growing confidence in the safety of plant-based compounds for biomedical applications, presenting these as low-toxicity alternatives to synthetic options [42].

Unfortunately, in developing countries, the implementation of circular economy practices, including the valorization of food by-products, faces challenges such as insufficient technological expertise, lack of public awareness, and weak enforcement of existing environmental laws. Although there is growing interest in turning vegetal by-products into valuable products, the overall lack of regulation and research impedes the country’s ability to fully embrace circular economy benefits [18].

## 4. The Possible Use of Plant By-Products in Biomedicine

### 4.1. Antimicrobial Agents

Over recent years, it has become well known that many microorganisms have developed resistance to a wide range of antibiotics, antifungals, and antiparasitics. Antimicrobial resistance (AMR) is a global public health crisis that threatens the effective prevention and treatment of a great number of infections caused by bacteria, viruses, fungi, and parasites [43,44].

#### 4.1.1. Antibacterial Activity

Bacteria acquire and express resistance genes and then share those genes with other bacteria driving the resistance phenomenon (bacterial selection). The threat is most acute for synthetic antibacterial agents, which explains the strong interest in biologically active plant compounds as potential alternatives. In contrast to synthetic agents, plant-based extracts often contain dozens or even hundreds of antimicrobial constituents acting synergistically. This complex chemical diversity not only enhances antimicrobial efficacy but also reduces the likelihood of bacteria developing simultaneous resistance to all active components, thereby offering a more robust and sustainable approach to combating microbial infections [44,45].

Diverse studies from 2016 to 2020 (Table 1) concluded that different vegetable organs accumulate promising compounds with antimicrobial properties. What is more, vegetal residues such as peels, pulps, and leaves from pomegranate, orange, yellow lemon, ginger, onion, potato, garlic, tomato, and banana have demonstrated their effectiveness against bacterial infections caused by both Gram-negative (*Escherichia coli*, *Pseudomonas aeruginosa*, *Klebsiella* spp., *Salmonella* spp., *Shigella sonnei*) and Gram-positive (*Streptococcus* spp., *Enterococcus faecalis*, *Bacillus* spp., *Staphylococcus* spp.) microorganisms, some of them able to cause major diseases difficult to treat [23,46,47,48,49,50,51]. Special attention should be paid to *Staphylococcus aureus* among these bacteria, because it is one of the most widespread bacterial pathogens. It causes a large number of skin infections and severe and invasive infections like pneumonia or endocarditis. *S. aureus* infections are particularly problematic due to their high prevalence, with the methicillin-resistant *S. aureus* (MRSA) the most important from the clinical point of view since it is a strain resistant to commonly used antibiotics, such as penicillin and methicillin [52]. Similarly, the number of *E. coli* strains resistant to commonly used antibiotics, such as amoxicillin or ciprofloxacin, is increasing, thus creating an urgent need to explore new treatment options, taking into account that the most common disease caused by this strain is the urinary tract infection [53].

Table 1 summarizes the results of several studies focused on testing the effect of extracts obtained from different vegetal residues against diverse bacteria able to cause human diseases. The effectiveness of plant by-products as antimicrobial agents, however, can depend on several factors, including the plant variety and the chemical used to extract the bioactive compounds.

Plant variety was determinant in the case of pomegranate since the results differed when used peels from sweet, sour, or bittersweet varieties [23]. The best results were obtained with the bittersweet variety Piñón Tierno de Ojós 8 (PTO8) and with the acidic variety Hicaznar (HIC) (Table 1). The skin extracts from the PTO8 variety exhibited Minimum Inhibitory Concentrations (MICs) of 1.5 μg mL^−1^ and 68.5 μg mL^−1^ against *B. subtilis* and *S. aureus*, respectively. Moreover, MICs reached values of 12.5 μg mL^−1^ and 21.3 μg mL^−1^ against *E. faecalis* and *Salmonella enterica*, respectively, when applied to skin extracts of the HIC variety. This variety behaved as the most effective against *S. aureus*, achieving MIC values of 57.2 μg mL^−1^. In the case of tomato, Tiny Tim variety appeared as the most effective against *S. aureus* (MIC = 0.625 mg by-product mL^−1^), *B. subtilis* (MIC = 1.25 mg by-product mL^−1^), *Listeria monocytogenes* (MIC = 2.50 mg by-product mL^−1^), and *Klebsiella pneumoniae* (MIC = 2.50 mg by-product mL^−1^) among a total of six tested varieties (Table 1). This high effectiveness has been ascribed to the high levels of carotenoids and phenolic compounds (like ellagic acid and punicalagins) in the Tiny Tim variety. These compounds are known for their ability to disrupt microbial cell walls, membranes, or enzymatic activities [49].

As previously mentioned, the type of extractant can condition the antimicrobial activity displayed by a given plant residue. However, in some cases, high and similar antimicrobial activity has been found using different extractants, as in the case of ethanolic and aqueous extracts of banana fruit peels against multidrug-resistant bacteria [51]. In contrast, when researchers used banana leaves [48], a large difference was observed between methanolic extraction, which was highly effective, and extraction with hexane, without an antibacterial effect. Similarly, in most cases, distilled water was a more appropriate solvent than methanol for lemon and orange peels as antibacterial agents, except for *K. pneumoniae*, which behaved sensitively when researchers used methanolic extracts of lemon peel [50].

In a study conducted by Naqvi et al. [47], the antibacterial effects of ginger, garlic, onion, and potato peels were tested against four bacterial strains: *E. coli*, *Bacillus cereus*, *B. megaterium*, and *S. aureus*. The results showed that ginger peels exhibited the strongest inhibition against all bacterial strains, while onion peels did not show any inhibitory effect [47]. In a separate study, coffee mucilage was found to have a growth-inhibitory effect against *B. cereus* [55]. This residue, rich in chlorogenic acid, likely acts through membrane disruption, acidification of the bacterial cytoplasm, and possibly interference with cell wall synthesis. The mechanisms by which these compounds exert antimicrobial effects vary notably between Gram-positive and Gram-negative bacteria due to differences in their cell wall structures. Gram-positive bacteria (as *B. cereus*) possess a thick peptidoglycan layer, which can be targeted directly by certain phenolic compounds causing membrane destabilization and enzyme inhibition. Conversely, Gram-negative bacteria have an additional outer membrane composed of lipopolysaccharides, which acts as a barrier to many compounds, requiring antimicrobial agents to penetrate or disrupt this layer to exert effects [55,56]. Some plant-derived compounds, such as terpenes, have lipophilic properties that facilitate disruption of the outer membrane, increasing permeability and allowing other active substances to enter the bacterial cell. This differential mode of action highlights the importance of understanding bacterial physiology when developing plant-based antimicrobials [57].

In an attempt to characterize the molecule(s) responsible for the antibiotic effect, after a first extraction, fractions were separated and tested for whether the antimicrobial activity differed between those fractions and the original pure extract [46]. In numerous cases, the key bioactive compounds with antimicrobial activity belonged to the phenolic compounds. In fact, frequently, there was a positive relationship between the antioxidant and antimicrobial activities and the phenolic content [23,48,49]. Similarly, essential oils, such as those from the seed of *Nigella sativa*, are rich in terpenes and phenolic compounds, which are known to exhibit strong antimicrobial properties. In the case of *N. sativa* seed oil, when used alone, it showed promising antimicrobial effects. However, when combined with conventional antibiotics like augmentin, oxacillin, and cephalosporin, the bacterial inhibition was more rapid and stronger than when either *N. sativa* or the antibiotics were used individually. This suggests a synergistic effect, where the combination of *N. sativa* with antibiotics could help overcome bacterial resistance by potentially disrupting the bacterial cell wall. This disruption may enhance the antibiotics’ effectiveness, offering a potential solution to combating bacterial resistance through synergistic therapeutic strategies [54,58].

However, despite the promising antimicrobial potential of plant extracts, a major challenge lies in standardizing these complex mixtures to ensure consistent efficacy and safety. The variability in plant composition due to factors like geographic origin, harvest time, and extraction methods complicates reproducibility. Regulatory agencies face difficulties in approving plant-based antimicrobials because of these inconsistencies, lack of clear active ingredient identification, and the absence of standardized potency metrics. Potential solutions include the development of advanced analytical techniques for comprehensive chemical profiling, establishing reference standards, and harmonizing guidelines across jurisdictions. Moreover, combination therapies, as demonstrated with *N. sativa* and antibiotics, may accelerate regulatory acceptance by leveraging known pharmaceutical agents to enhance efficacy and reduce resistance risk [59].

In any case, there are some examples of patents related to the antimicrobial effect of plant by-products. For example, patent WO 2020/230044 A1, owned by the University of Parma (Italy) (https://www.lens.org/lens/patent/084-002-100-013-121/frontpage?l=en, accessed on 11 June 2025), describes the production of antimicrobials from vegetable waste against *S. aureus*, *L. monocytogenes*, *Bacillus cereus*, *Salmonella* spp., and *E. coli*, among others.

**Figure 5:**
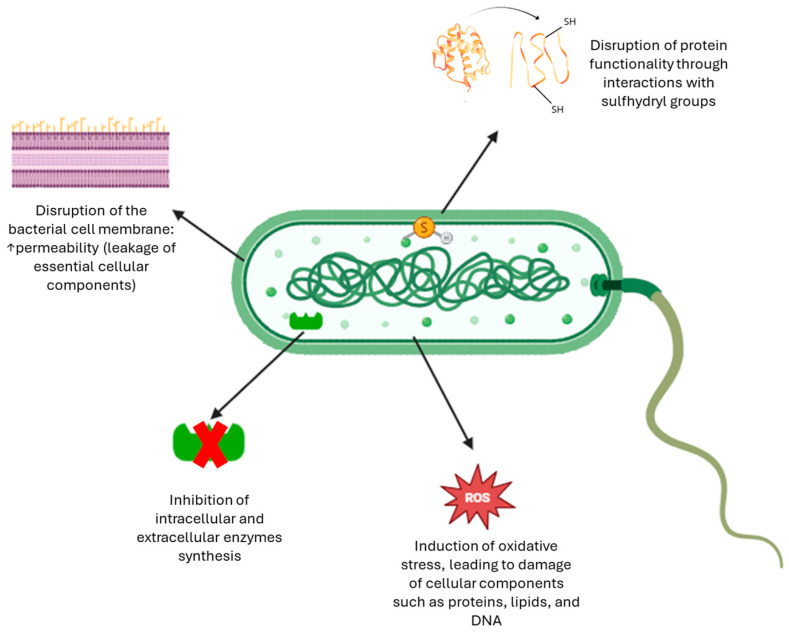
Antibacterial mechanisms of phenolic compounds [56].

To sum up, Figure 5 illustrates the multifaceted antimicrobial effects of phenolic compounds, including their modes of action on bacterial membranes and intracellular targets [56].

#### 4.1.2. Antifungal Activity

Fungal infections have increased over the last decades. According to the Global Action Foundation for Fungal Infections, about 30 fungal species cause 99% of human fungal diseases, producing more than 1.6 million deaths each year in the world. Some fungal diseases are acute and severe, such as cryptococcal meningitis; others are recurrent, such as candida vaginitis or oral candidiasis in acquired immunodeficiency syndrome; and others become chronic, such as pulmonary aspergillosis or tinea capitis [60,61].

The uncontrolled use of antifungals, similar to what happens with antibiotics, coupled with the rapid adaptability of fungi to change, has led to decreased efficacy of pharmacological treatments as a consequence of the increased fungal resistance [62]. In addition, according to the World Health Organization (WHO), there are currently only four classes of antifungal drugs available and very few candidates in clinical development, which makes it urgent to find alternatives, including the potential use of plant residues as antifungal agents [63]. Table 2 presents the results of four studies focused on analyzing the efficacy of different organs (leaves, rhizomes, bulbs, fruits) of seven plants against four genera of fungi (*Aspergillus*, *Fusarium*, *Trichophyton*, *Penicillium*) which can be harmful to human health, either by causing a specific disease, such as keratitis or ringworm, or by producing and releasing toxins that can reach the consumer through the food chain [64].

The efficacy of plant residues as antifungal agents, however, depends on the extract fraction [51]. Pomegranate extracts [23], especially those obtained from the bittersweet variety PTO8, were highly effective in inhibiting the radial growth of *Fusarium verticillioides* (Table 2). The highest value of inhibition was achieved with the n-butanolic fraction (radial growth inhibition of 70%), whereas the hexane fraction with the highest polyphenol concentration and antioxidant activity surprisingly did not show the highest antifungal activity. The authors suggested that the n-butanolic fraction might contain antifungal compounds other than those usually reported in pomegranate peel extracts (punicalagins and ellagic acid). Considering that *F. verticillioides* is resistant to most of the antifungals currently used, such as azoles or amphotericin B, these results are promising in antifungal therapy [67]. In a similar way, banana leaf extracts applied in certain concentrations could be even more effective antifungal agents than nystatin, an antifungal drug currently used in medicine [48]. Banana leaf extracts likely contain tannins, flavonoids, saponins, phenolic acids, and alkaloids, which act together to inhibit fungal growth. Tannins disrupt cell walls, flavonoids reduce enzyme activity, saponins damage membranes, phenolic acids alter metabolism, and alkaloids may inhibit cell division, enhancing the antifungal effects of the extract. Moreover, ginger, garlic, and potato peel extracts showed medium positive inhibition against *F. verticillioides*, while onion peels exhibited slight positive inhibition [47]. This effect could be due to the presence of bioactive compounds such as gingerol [68,69] in ginger and allicin in garlic, which demonstrated antifungal properties [70]. In a clinical study, allicin was administered intravenously to mice before and after *Trichosporon asahii* infection. The results showed that allicin significantly improved survival and reduced the fungal burden in kidneys, the spleen, and the liver. Further analysis revealed that allicin affected fungal plasma membrane integrity by downregulating ergosterol biosynthesis genes and disrupting the cell wall by inhibiting glucan and chitin synthase. Additionally, allicin induced oxidative stress and mitochondrial dysfunction, leading to an energy deficit in the fungus, contributing to its antifungal effects [71].

The efficacy in vitro of aqueous extracts of mandarin and banana peels was tested in inhibiting the growth of mycotoxin-producing strains of fungi: *A. flavus*, *A. tubingensis*, and *P. griseofulvum* [65]. While mandarin peel appeared very effective in reducing the growth of *P. griseofulvum* (by around 90%), banana peel decreased the growth of *P. griseofulvum*, *A. flavus*, and *A. tubingensis* by 20%. This effect was dose-dependent, since an increase in the concentration of the residue from 5% to 10% (*w*:*v*) led to the percentage of inhibition for *A. flavus* reaching 80% with the mandarin residues and 40% with banana ones [66]. Additionally, a study published in 2024 achieved 80% inhibition of *A. flavus* growth using banana peels [72]. However, the results were not always positive with vegetable by-products: neither kiwi peel nor a mixture of mandarin and banana showed any inhibitory effect against those mycotoxin-producing fungi. Some studies attributed the antifungal activity to the cinnamic acid present in plants, the compound that inhibits the fungal cytochrome P450 CYP53A15, whose role is preventing the demethylation of lanosterol, a triterpenoid precursor of the sterol that makes up the fungal membrane [73].

While many studies have explored the antifungal activity of plant residues, few have focused on understanding the underlying mechanisms of action of these plant by-products. This lack of mechanistic insight makes it challenging to fully evaluate their potential as antifungal agents. Recent research has examined the antifungal potential of grape pomace extract (GPE), a by-product from wine production. Studies focusing on GPE’s effects on fungi, particularly the *Saccharomyces cerevisiae* yeast model, have identified key targets involved in its antifungal activity. GPE has been shown to disrupt the ergosterol biosynthesis pathway by inhibiting key enzymes, leading to the accumulation of toxic sterol intermediates and a compromised cell membrane. Additionally, GPE’s action in apoptosis regulation suggests that it may induce programmed cell death in yeast, although without the involvement of the Yca1 protein, which is typically a key regulator of apoptosis in *S. cerevisiae*. Moreover, GPE affects the cell wall integrity, particularly by interfering with the protein kinase C (Pkc1) signaling pathway, which is essential for maintaining cell wall structure and function [74].

Another study focused on polymethoxylated flavonoids (PMFs) from *C. reticulata* peel reveals complementary antifungal mechanisms. PMFs disrupt fungal cell membrane integrity by altering ion permeability, causing potassium (K^+^) to exit and sodium (Na^+^) to enter cells, leading to osmotic imbalance and cellular ageing. Additionally, PMFs impair cell wall strength by inhibiting chitin synthesis, a key structural component, resulting in weakened hyphae and cell lysis [75].

These findings provide valuable insights into how plant by-products may interfere with critical cellular processes in fungi, offering potential for the development of new antifungal agents targeting multiple pathways simultaneously.

#### 4.1.3. Antiparasitic Activity

Parasitic infections are a global public health problem due to their high frequency in developing countries, their introduction into developed countries through people migration and travels, and their high morbidity [76]. The inexistence of vaccines to combat most of these diseases, and the fact that some drug treatments are becoming ineffective as a consequence of widespread resistances, underscore the necessity of prevention and finding new treatment strategies [77].

Cryptosporidiosis is responsible for 0.6–7.3% of diarrheal diseases in countries with modern sanitation systems and for an even higher percentage in areas with poor sanitation [78,79]. Current treatments are self-limiting, so as a new approach, some studies have tested plant by-products against this protozoo (Table 3). Pomegranate peel has proven to be effective against *Cryptosporidium parvum* in a murine model, with a reduction in oocyst shedding (oocysts 0.01 g^−1^ feces) of up to 100% [80]. Moreover, ethanolic extract from olive pomace has been demonstrated to be effective in a WST-1 assay against *C. parvum* (MIC = 250–500 µg mL^−1^). However, in this study, other plant by-products such as grape seed extract peels did not show any significant activity against this protozoo (MIC = 500–1000 µg mL^−1^) [81].

The protozoan parasite *Trichomonas vaginalis* is the causative agent of trichomoniasis, one of the most common non-viral sexually transmitted infections in humans. In contrast, *T. foetus* targets the gastrointestinal system of livestock, leading to trichomonosis, a condition marked by diarrhea and digestive disturbances [82]. The peel powder obtained from *S. lycopersicum* var. *cerasiforme* was effective in inhibiting the growth of different strains of human and animal trichomonas, achieving percentages of growth inhibition up to 70% for *T. vaginalis* G3, 70% for *T. foetus* C1, and 80% for *T. foetus* D1 [83].

**Table 3:** Antiparasitic effects of plant residues.

Plant	By-Product	Parasite Specie	Type of Extractant	Disease	Reference
Olive (*Olea europaea*)	Olive pomace	*C. parvum*	Deionized water, 70% aqueous ethanol or heptane	Criptosporidiosis	[81]
Pomegranate (*P. granatum*)	Peel powder		Aqueous		[80]
Cherry tomato (*S. lycopersicum* var. *cerasiforme*)	Peel powder	*Trichomonas* spp.	Dimethyl sulfoxide/water (1:1)	Trichomoniasis	[83]
Orange (*C. sinensis*)	Leaves	Promastigote forms of *L. amazonensis*	Hexane, ethyl acetate, dichloromethane/ethanol (1:1) or ethanol/water (7:3)	Leishmaniasis	[84]

Leishmaniasis is among the top ten neglected tropical diseases (NTDa) with the highest incidence in the world. The disease is manifested in three main forms: visceral, cutaneous, and mucocutaneous. *Leishmania amazonensis* is associated with diffuse cutaneous leishmaniasis, a rare and severe form of the disease characterized by a widespread invasion of the skin and the gradual development of diverse lesions, including small, raised bumps, nodules, and thickened plaques. According to the WHO, this type of leishmaniasis is difficult to treat [85,86]. The antileishmanial potential of extracts obtained with dried leaves of *Citrus sinensis* demonstrated effectiveness against *L. amazonensis* promastigotes and intracellular amastigotes, with the hexane extract being the most effective (IC50 = 25.91 and 39.78 µg mL^−1^, respectively, against promastigotes and amastigotes) [84].

However, as commented previously for the potential antibacterial activity of vegetable residues, further research is needed to identify the active compounds in all of these by-products to gain deeper knowledge of the mechanism of action and to increase the therapeutic range. Moreover, considering the wide variety of parasitic infections and the lack of effective treatments, plant residues appear a promising tool for antiparasitic therapies.

### 4.2. Antitumoral Activity

Cancer is still one of the world’s principal causes of morbidity and mortality. The International Agency for Research on Cancer estimated that in the year 2020, approximately 19.3 million new cases of cancer would be diagnosed worldwide and the number of new cases will increase to 28 million per year by 2040 [87]. The malignancy of tumors poses a significant threat to an individual’s health. The prognosis for many patients with malignant tumors remains limited, largely due to the lack of highly effective therapeutic options [88].

In the history of natural compounds used in cancer therapy, ones that deserve special mention are the Vinca alkaloids, vincristine and vinblastine, which inhibit microtubule polymerization by binding to tubulin, causing cell cycle arrest in the metaphase, and triggering apoptosis, as well as the alkaloid paclitaxel (also known as Taxol^®^, Research Triangle Park, NC, USA), extracted from the bark of *Taxus brevifolia*. It stabilizes microtubules, preventing their depolymerization, which disrupts mitosis. It also triggers cell cycle arrest at the G2/M checkpoint, ultimately leading to programmed cell death [89]. In addition, emerging research highlights that many natural compounds exhibit cancer-selective properties, demonstrating antiproliferative, pro-apoptotic, and cytotoxic effects, specifically against malignant cells [90]. As an example, hesperetin, typically found in citrus peels, exerts its anticancer (colorectal cancer) effects by scavenging reactive oxygen species generation (ROS) and enhancing the activity of antioxidant enzymes like superoxide dismutase, glutathione reductase, and glutathione-S-transferase. Hesperetin also modulates the expression of key proteins such as TGF-β1, which regulates cell cycle progression and apoptosis. Additionally, it induces apoptosis by upregulating pro-apoptotic proteins like p53 and downregulating antiapoptotic proteins, while reducing the expression of the proliferation marker Ki67. These molecular actions collectively contribute to its anticarcinogenic properties [91]. Furthermore, the essential oils from the leaves of *Vitex macrophylla* exert their antitumoral effects (breast cancer) by interacting with various molecular pathways. The main component, citral, induces apoptosis by causing mitochondrial membrane depolarization, which leads to the release of cytochrome c and activation of caspases, triggering the intrinsic pathway of apoptosis. Additionally, citral disrupts microtubule dynamics by binding to tubulin, inhibiting its polymerization and leading to cytoskeletal destabilization. This results in alterations to the actin cytoskeleton, impairing cell motility and proliferation. Moreover, the essential oils regulate oxidative stress by increasing the production of ROS, which further contributes to cellular damage and death. These oils also exhibit chemopreventive properties by modulating signaling pathways such as AMPK/mTOR and by suppressing the activation of pro-inflammatory cytokines like TNF-α, contributing to the inhibition of tumor progression [92].

In that context, novel compounds obtained from vegetal residues are of growing interest. In fact, mango seed kernels, citrus peel oils, grape seeds and leaves, and pomegranate, apple, avocado, tomato, and potato peels have shown in vitro activity as potential antitumoral or chemopreventive agents (Table 4) [93,94,95,96,97,98,99,100,101,102,103,104,105,106,107].

The extraction method of bioactive compounds is important and should be optimized, considering factors such as the type of solvent, fractions, solubility, or temperature. In a study with mango seed kernels, green pressurized-liquid extraction and dynamic maceration were used to evaluate in vitro bioactivity as antitumoral. Both evidenced antiproliferative activity against the human colon adenocarcinoma cell line HT-29; however, green pressurized-liquid extraction was more effective. Mangiferin is the major component in the sugar of mango seed kernel and exhibits significant antiproliferative activity through several molecular mechanisms: it protects cells from oxidative stress and DNA damage, downregulates inflammation, and induces apoptosis by inhibiting NF-κB activation. Additionally, mangiferin triggers cell cycle arrest and reduces proliferation in malignant cells. Other compounds like gallic acid and ellagic acid further enhance the extract’s effects by modulating apoptosis and cell cycle progression and inhibiting signaling pathways such as NF-κB and Akt. The synergistic interactions of these compounds contribute to the overall potent antiproliferative activity observed [93]. Efforts have been made to explore the therapeutic potential of mangiferin through clinical trials. However, its clinical use remains limited due to its low water and fat solubility, restricted absorption, and poor bioavailability, which hinder its oral efficacy. While trials have attempted to assess its benefits, these challenges have slowed progress in realizing its full potential. To address these issues, researchers are focusing on developing specific formulations to enhance its oral bioavailability, aiming to improve its clinical applications and effectiveness in treating various conditions [108].

The agri-food industry generates tons of processing by-products that are being accumulated on the soil in adjacent areas. As an example, pomegranate peels [99] are the major by-products of the processing of pomegranate juice. A DPPH assay demonstrated that this peel has bioactive compounds (β-glucans) with antioxidant activity. Recent studies have reconfirmed their antitumoral properties [100]. Pomegranate peel compounds, such as ellagic acid and punicalagins, inhibit the STAT3 pathway, which regulates processes like angiogenesis, cell proliferation, and apoptosis, often dysregulated in cancer cells. Additionally, pomegranate compounds target the PI3K/Akt pathway, reducing cell survival and proliferation by inhibiting key proteins like Akt and mTOR. These actions help prevent tumor growth, metastasis, and resistance to cell death, making pomegranate a potential natural agent for cancer prevention and treatment [109,110,111]. The results from pre-clinical studies on pomegranate have been so promising that they have advanced to clinical trials. In clinical studies, pomegranate has been tested primarily in prostate cancer patients. They have measured the impact of pomegranate juice on oxidative stress biomarkers, such as 8-hydroxy-20-deoxyguanosine (8-OHdG), and its ability to delay the increase in prostate-specific antigen (PSA). One study showed that drinking pomegranate juice (equivalent to 570 mg of polyphenol gallic acid per day) after prostate surgery increased the mean time for PSA levels to double from 15 months to 54 months. These results are among the key findings that suggest pomegranate’s potential in prostate cancer treatment [111].

A study focused on grape seed proanthocyanidin extract [97] showed that this extract attenuated doxorubicin-induced ROS and prevent the cardiotoxicity of that antineoplasic due to its antioxidant capacity. Similarly, citrus peels, abundant in compounds with antioxidant activity, provide an additional shield against oxidative stress, thereby enhancing the overall cancer preventive and protective capacity within the body [94]. These natural sources could also be explored as adjuvant treatments in therapeutic settings, though this approach warrants further investigation through animal studies and subsequent clinical trials.

In other scenarios, with apple peel [101] and avocado seed [103], flavonoids and triterpenoids, respectively, displayed superior properties in not only antioxidation but also antitumor aspects, leading to the apoptosis of tumor cells. Likewise, diverse active compounds extracted from grapevine leaves have shown antihyperglycemic, antioxidant, anti-inflammatory, analgesic, and antipyretic properties [112]. In addition, in vitro studies have demonstrated effective cytotoxic activity of grapevine leaves against lung [113], breast [114], and leukemia [115] cancer cells. This effectiveness, however, can vary among grapevine cultivars and it is modulated by biotic and abiotic factors affecting this crop: elevated air temperatures and the association of grapevines with mycorrhizal fungi can enhance the cytotoxic activity of grapevine leaf extracts because both factors can induce the accumulation of phenolic compounds [95].

These promising results obtained in in vitro assays have recently been supported by preclinical studies performed with mice [102]. A nanoemulsion of carotenoids extracted from sweet potato peel reduced both the size and weight of tumors, although the inhibition efficiency of tumors in the animals was dependent on the type of carotenoid, the preparation method of carotenoid nanoemulsion, the dose applied, the administration length, and the cancer cell type. It was hypothesized that the effective inhibition of MCF-7 breast cancer cells could occur through a passive targeting effect and that the antitumor efficiency could be due to the synergistic effect of β-carotene and other carotenoids present in the nanoemulsion. These hopeful results emphasize the necessity of research in more advanced clinical phases. Building upon the compelling evidence of antitumoral activity in fruit and vegetable by-products, some studies delve deeper into their mechanisms of action (Table 4). That is the case of a study focused on Brazilian native fruits like açaí and camu-camu [116]. The diverse by-products of these fruits have been explored using different extractants, as shown in Table 4, each yielding distinct bioactive profiles and antitumoral activities. The hydroethanolic extract of açaí seeds demonstrated significant antitumoral effects on A549 lung cancer cells (dosage and period = 1.25–200 µg mL^−1^ for 48 h) by increasing antioxidant activity, inducing apoptosis, and halting the cell cycle in the G0/G1 phase, ultimately reducing cell viability by 72.07% [104]. The aqueous extract of açaí seeds also exhibited strong growth inhibition against NCI-H460 lung cancer cells (GI50 = 22 µg mL^−1^) while maintaining safety for normal cells (dosage and period = 0–125 μg mL^−1^ for 48 h) [105]. For camu-camu, its hydroethanolic seed extract (dosage and period = 100–900 μg mL^−1^ for 48 h) proved highly effective in reducing ROS generation and cisplatin-induced mutagenic damage. It selectively inhibited A549 lung cancer cell growth (GI50 = 251 µg mL^−1^) without harming normal cells, underscoring its dual antioxidant and cytotoxic capabilities [106]. The camu-camu seed water–ethanol–propanone extract (dosage and period = 100–900 μg mL^−1^ for 48 h) exhibited antioxidant, cytotoxic, and antiproliferative effects on the A549 lung cancer cell line (IC50 of 581.1 µg mL^−1^). Camu-camu’s anticancer effects are primarily driven by its polyphenolic compounds, such as methylvescalagin, proanthocyanidin A2, ellagic acid, and epicatechin. These compounds reduce reactive ROS generation, inhibit cancer cell proliferation, and induce apoptosis in cancer cells. They also possess anti-inflammatory properties, reducing TNF-α release and inhibiting NF-κB activation [107]. Examples of patents related to the use of fruit by-products as antitumor agents include US 2005/0147723 A1, which is owned by the Cornell Research Foundation (USA) (https://www.lens.org/lens/patent/027-550-960-192-801/frontpage?l=en, accessed on 11 June 2025), and CN 114558053 A, which is owned by the Hospital for People in the Jimo District of Qingdao City and the Qingdao University of Science and Technology (China) (https://www.lens.org/lens/patent/006-339-009-282-720/frontpage?l=en, accessed on 11 June 2025). The former, titled ‘Apple peel powder, methods of making, and uses thereof’, outlines a procedure for treating cancer in patients through the administration of apple peel powder. Cancer cell proliferation can also be avoided by bringing the cells into contact with the powder under conditions that effectively inhibit it. The second patent, titled ‘Waste fruit peel extract composition with effect of treating gastric cancer’, demonstrates the effectiveness of a mixture of guava, annona squamosa, and passion fruit peel extracts against gastric cancer.

### 4.3. Dermocosmetic Applications

Due to the growing interest in eco-labeled cosmetics, the use of biomolecules identified and isolated from plant residues opens up new perspectives on their sustainable application, contributing to the circular economy. In this line, almost a decade ago, it was proposed that we pursue the use of polyphenols obtained from vegetal residues in the cosmeceutical industry as chemopreventive agents for skin disorders because they possess anti-inflammatory, immunomodulatory, and antioxidant properties [25].

#### 4.3.1. Photoprotection

The correct use of photoprotection can attenuate and mitigate skin damage caused by different types of radiation such as UV, HEVL, or IR-A [117]. Studies have demonstrated the efficacy of naturally occurring polyphenols, such as oleuropein from olive leaves, resveratrol from grape skin, or proanthocyanidins from grape seeds, against UV radiation-induced inflammation, oxidative stress, and DNA damage. Most of the natural polyphenols are pigments and they can absorb UV radiation, including the entire UVB spectrum and part of the UVC and UVA spectra [117].

Olive leaves, a residue produced during olive harvesting and tree pruning, are rich in polyphenolic compounds. Among these, oleuropein (OLE) is the most abundant and well-studied constituent, known for its effective photoprotective, antimutagenic, and antioxidant properties [118]. When comparing this OLE extract with the positive control of the study, resveratrol (RES), the results were very similar in terms of antioxidant activity (OLE EC50 =11.75 μg mL^−1^ and RES EC50 = 12.64 μg mL^−1^). When in vitro sun protection factor (SPF), UVA/UVB ratio, and λc (nm) values of developed formulations containing OLE, *Polypodium leucotomos* (PLE), and RES were studied, it was found that OLE showed a significant increase in SPF values from 22 for the control fraction to 42 and 56 for the concentrations of 3 and 5% of OLE. The results obtained for OLE were promising and more effective than those of the PLE extract. The antiphotomutagenesis effect was evaluated upon CD138 (*ogg1*) strain irradiation with solar simulated light (SSL). RES treatment led to an approximate 2.9-fold reduction in the number of SSL-induced CanR mutants compared to the control, while OLE achieved a 2.4-fold decrease. Notably, only OLE demonstrated effective photoprotective activity following SSL exposure lasting longer than two hours. In conclusion, oleuropein appeared the most effective active agent due to the combination of the three effects. These results can be used to formulate sunscreens or solar nutricosmetics containing this OLE extract, not only preventing immediate damage such as sunburn but also providing protection for DNA damage or skin ageing. It is an underexplored, promising, and sustainable compound [118].

#### 4.3.2. Antiageing

Skin ageing is manifested in clinical features such as wrinkles, hyperpigmentation, or sagging skin as a result of changes in the functionality of skin components at the molecular level [118]. This is the case, for example, of elastase, an enzyme that hydrolyses elastin, a connective tissue protein that provides elasticity to tissues. High activity of this enzyme causes loss of the skin firmness and elasticity [119]. The same occurs with tyrosine, which is responsible for the synthesis of melanin in melanocytes, but, when there is an overproduction of melanin in the skin, pigmentary disorders occur, such as age spots and melasma. Numerous skin routines include antiageing products containing natural actives such as polyphenols [120].

In the agri-food industry, large quantities of by-products are generated, with the wine industry being one of the most important. Seeds, pulp, skins, leaves, stems, and wine lees contain bioactive compounds of interest, including polyphenols, oils, or lignins. Grape stem extracts inhibited tyrosinase and elastase, with this activity especially significant for extracts obtained from the Syrah grapevine variety: the inhibition percentages reached 53.83% and 98.02% for tyrosinase and elastase, respectively [121]. Therefore, these results support the idea that this by-product can be applied in cosmetic products to combat wrinkle and skin pigmentation disorders. Moreover, after applying shoot extracts of *Thymus pulegioides*, the tyrosinase activity was inhibited [122]. This herb was proposed as a functional food ingredient with important antioxidant, antiproliferative, and neuroprotective effects based on its content and profile of phenolic compounds.

#### 4.3.3. Commercial Insights

The commercial landscape for plant-based dermocosmetic products has seen significant growth in recent years, driven by consumer demand for natural and sustainable alternatives. Unlike pharmaceutical products, dermocosmetics benefit from a less stringent regulatory framework, facilitating faster market entry and innovation [123]. However, ensuring the safety and efficacy of these products remains a critical challenge. As highlighted in recent regulatory reviews, including the 2013 EU Cosmetics Regulation, rigorous safety assessments must still be conducted, especially when using novel or less-characterized natural ingredients sourced from plant by-products. Moreover, there is a need for improved post-market surveillance and consumer reporting of adverse effects to fully understand the safety profile of these emerging dermocosmetic formulations [123].

Many companies are capitalizing on the bioactive properties of plant residues and by-products to develop formulations with antioxidant, antimicrobial, and anti-inflammatory benefits. Famous brands like Caudalie^®^ (Bordeaux, France)^.^ and Apivita^®^ (Athens, Greece)^.^ utilize grape seed extracts (an abundant winery residue) in several of their products, capitalizing on its rich polyphenol content to provide antioxidant protection and skin rejuvenation [124]. An illustrative example of the valorization of agricultural residues in the dermocosmetic sector is the Antcare project, part of the BIO4Africa technology catalogue. This initiative focuses on the upcycling of organic apple by-products, including seeds and peels from organic apple juice production, to create natural cosmetic ingredients. Through a sustainable process powered by renewable energy and employing recycled and recyclable packaging, the project produces apple paste rich in nourishing and antioxidant compounds, exemplifying circular economy principles and sustainable innovation in cosmetics [125,126].

However, despite the widespread use of botanical ingredients in the dermocosmetic industry, with brands such as Kiehl’s^®^ (Paris, France), Origins^®^ (Cambridge, MA, USA), L’Occitane^®^ (Manosque, Provence, France), and Weleda^®^ (Arlesheim, Suisse) incorporating plant-based ingredients, only a small fraction of these compounds are actually derived from agricultural or food-processing residues. Most commercial formulations still rely on cultivated or wild-harvested plant materials, thus missing the opportunity to valorize large volumes of bioactive-rich by-products.

In addition, various patents demonstrate the innovation potential of plant residues in dermocosmetics. For instance, patent CN117175533 discloses a combined plant extract for eliminating mites and acne, obtained through ultrasound-assisted water extraction and alcohol precipitation from sources such as *Nepeta cataria*, bamboo, celery seeds, and orange peels [127]. Similarly, patent CN100493711C discloses a cosmetic gel formulation with antioxidant, antiageing, and antibacterial effects that incorporates plant extracts and oils derived from botanical sources and plant residues, including grape seed oil [128].

In the coming years, plant residue-based dermocosmetics are expected to evolve beyond trend into a strategic pillar of sustainable innovation. However, to truly scale this model, the key will be fostering collaboration between the agri-food and cosmetic industries. By connecting producers of plant-based residues with companies specialized in green extraction and formulation, it becomes possible to transform by-products into high-value cosmetic ingredients. This approach not only reduces environmental impact but also creates economic value through circular economy principles, bridging sustainability and innovation in a tangible way [129]. To support this transition, strong and coherent regulatory frameworks will be essential, both to guarantee product safety and efficacy and to encourage the valorization of agricultural by-products through legal incentives, quality standards, and traceability requirements [130].

### 4.4. Other Applications

#### 4.4.1. Human Microbiota and Probiotics

**Figure 6:**
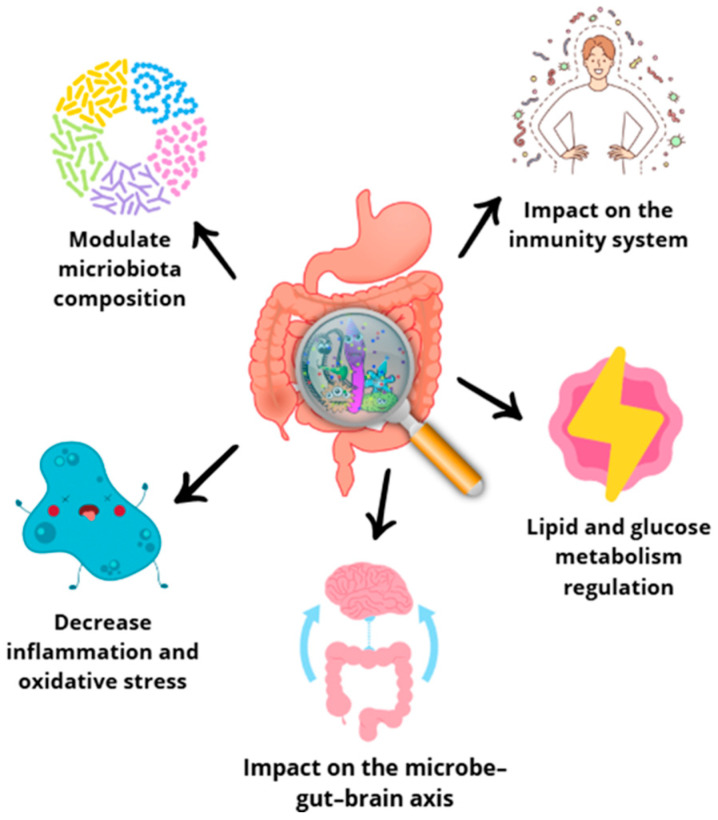
Effects of polyphenols on gut microbiota [117,118,119].

The human body hosts a remarkable diversity of microbes, constituting approximately 1–2% of its total weight [131]. Microorganisms colonize all surfaces of the human body, but the gut is the site particularly rich in microbiome, colonized by *Lactobacillus* spp., *Clostridium* spp., *Bacteroides* spp., *Prevotella* spp., and *E. coli* species. Diet assumes a predominant role in shaping the composition of the intestinal microbiota. The commensal microbiota actively participates in the fermentation of dietary components, which plays a crucial role in providing the host with essential nutrients and chemical signals that profoundly affect both immunity and metabolism (Figure 6). A noteworthy aspect is the presence of prebiotics among dietary substrates. Prebiotics are non-digestible food products that stimulate the growth of symbiotic bacterial species already present. This dynamic interplay between diet and the intestinal microbiota highlights the potential for dietary interventions to modulate microbial communities and support host health [132,133,134].

Polyphenol consumption may be related to the abundance and diversity of the gut microbiota [135]. Table 5 summarizes the conclusions of diverse studies [136,137,138], which have demonstrated that fruit peels from yellow watermelon, honeydew, papaya, orange, olive, apple, banana, and passion fruit exert beneficial effects on the human microbiota, as they contain a reasonably high amount of potential prebiotic compounds such as polyphenols (inulin, cellulose, hemicellulose, fructans, lignin, or pectin).

Recently, the polysaccharide extract obtained from yellow watermelon peel showed high efficacy in stimulating the growth of probiotics, specifically *Lactobacillus rhamnosus* and *Bifidobacterium bifidum*, and its effectiveness was higher than that shown by other peel extracts from honeydew and papaya. Furthermore, polysaccharide extracts from watermelon and honeydew peels demonstrated robust resilience under acidic gastric conditions and digestion by α-amylase in the small intestine. This resilience positions them as promising prebiotic candidates for further research and potential application in the development of functional foods and nutraceutical products [138].

The growing interest in this topic is attested to by the research projects that are being developed—among them, ‘Birbizi’, developed at the Public University of Navarra (Spain)—and based on good innovative practices for reusing whey and underused by-products from agroindustry. Among the vegetable residues worth mentioning are orange peel and aqueous juice obtained from the residue remaining after extracting oil from the olives. The final drinkable products obtained are rich in beneficial bacteria for the microbiota, vitamin C, and antioxidants [136]. In the same line, the addition of apple and banana fibers to fiber-enriched skim yogurts preserves the viability of various probiotic strains (*Bifidobacterium animalis* subsp. *lactis* Bl04, HN019, B94, and *Lactobacillus acidophilus* L10) for four weeks of cold storage. These fiber-enriched yogurts had enhanced levels of short-chain fatty acids and polyunsaturated fatty acids [137]. All these findings suggest the potential application of these fruit by-products in developing new probiotic yogurts with enhanced nutritional value.

#### 4.4.2. Sugar Replacement for Diabetics

The International Diabetic Federation Atlas [139] reports that 10.5% of the adult population from 20 to 79 years are diabetic and most of them (90%) suffer from type 2 diabetes, characterized by insulin resistance and hyperglycemia. Assays performed with diabetic rats fed with cupcakes made with either sucrose or a powder made from stevia leaves (SLP), banana peels (BPP), and carrot leaves (CLP) showed promising results. The diabetic rats fed with cupcakes including the vegetable residues showed an improved lipid profile in comparison with the group fed with sucrose-made cupcakes. Concretely, the consumption of cupcakes fortified with SLP, BBP, and CLP led to a decrease the levels of total cholesterol, glucose, triglyceride, glucose, and low-density lipoproteins (LDLs). This was particularly noticeable in cupcakes supplemented with a mixture of CLP and SLP. There was also a significant increase in high-density lipoprotein (HDL) levels for diabetic groups treated with BPP, CLP, SLP, and their mixtures. As diabetic people must avoid high-fat and high-sugar food, the substitution of sucrose by other vegetable residues may represent a good alternative to eating some sweet foods, avoiding the negative effects of sucrose [140].

A similar approach was taken in a recent clinical study using a nutraceutical derived from thinned nectarines (immature fruits discarded during agricultural thinning). These nectarines are rich in abscisic acid, a compound involved in glucose regulation. In a 12-week trial with type 2 diabetic patients, supplementation with the nectarine extract significantly reduced fasting blood glucose levels. Notably, the lower dose also decreased insulin levels by nearly 30%, suggesting improved insulin sensitivity. This highlights the potential of fruit residues not only as sugar substitutes but also as bioactive tools to support glycemic control in diabetic populations [141].

In addition, recent studies have explored the use of dried apple pomace as a sugar substitute in some sweet food products. The replacement of sucrose in chocolate with this plant-based residue significantly enhanced both the antioxidant activity and the total phenolic content of the product [142]. Similarly, biscuits enriched with 20% apple pomace exhibited a significantly lower glycemic index compared to control biscuits. These findings highlight how a common fruit residue can be valorized to simultaneously reduce the sugar content and enhance the functional antioxidant profile of sweet products, aligning with both health and sustainability goals [143].

#### 4.4.3. Tissue Engineering

In the field of tissue engineering, biodegradable polymers derived from natural and synthetic plant by-products have shown great potential for developing scaffolds that aid in tissue regeneration. Natural polymers that can be derived from agricultural by-products offer an eco-friendly alternative for scaffold materials. These by-products can be processed into polymers with desirable properties for promoting tissue growth and healing, making them an increasingly valuable resource in tissue engineering [144,145].

A key example is the use of lignin, a major component of plant cell walls. Lignin has antioxidant, antimicrobial, and immunomodulatory properties, making it useful in tissue engineering. It has been used in creating nanofibers, hydrogels, and other materials for regenerating bone, liver, kidney, and neural tissues. Additionally, lignin can improve the mechanical properties of scaffolds and promote the regeneration of specific cells, such as osteoblasts and hepatocytes [24].

Moreover, pectin extracted from citrus peel by-products has shown promise in hydrogel formation to support soft tissue regeneration. A study developed green hydrogels using pectin from orange peels, demonstrating their potential as carrier systems for analgesics and antibiotics, facilitating drug delivery and promoting tissue healing [146].

#### 4.4.4. Drug Delivery Systems

Drug delivery systems have significantly advanced the way medications are administered, providing targeted and controlled release to improve therapeutic outcomes and reduce side effects [147].

Among the materials used in these systems, plant-derived by-products offer a promising alternative to synthetic polymers. For example, polysaccharides such as cellulose, pectin, and starch are frequently used to form nanoparticles, microgels, and hydrogels for drug encapsulation. These polysaccharides can be extracted from various plant by-products, such as the peels of some fruits (citrus peels for pectin), woody biomass (for cellulose), and corn or potato by-products (for starch). These plant-based materials not only offer biocompatibility but also break down into harmless by-products like glucose or xylose, which are naturally metabolized by the body [8,24,148].

## 5. Economic and Technical Impact

**Figure 7:**
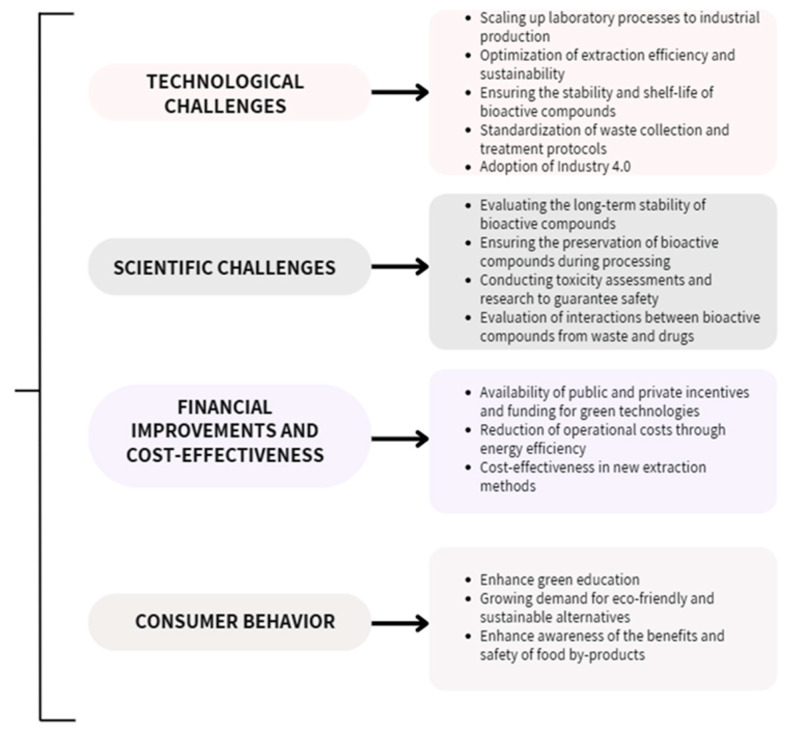
Technological and scientific challenges, economic impacts, and consumer attitude involved in the valorization of plant by-products [149].

The valorization of vegetal by-products offers substantial economic benefits by transforming plant by-products into valuable resources, aligning with sustainability and circular economy principles. However, the process faces significant technical challenges, such as optimizing extraction methods and ensuring product quality at scale. Addressing these challenges requires investment in research, infrastructure, and efficient energy use to achieve cost-effectiveness and widespread commercial success. The authors have presented their perspective on these challenges and have summarized these key aspects in a comprehensive figure (Figure 7) [149].

From an economic perspective, the successful valorization of food by-products is closely tied to the financial viability of these practices. While European consumers are increasingly inclined toward greener alternatives, the acceptance of items derived from food by-products has historically been limited. However, this trend is gradually evolving due to the strengthening of food safety regulations [40,41] and an increasing number of research studies and toxicity assessments that enhance consumer trust [42]. Thus, economic incentives and funding opportunities are critical in accelerating this transition and fostering investment in green technologies. These financial supports, alongside increasing consumer acceptance and trust in the benefits and safety of by-product-derived items, are expected to drive higher demand and facilitate market integration. In the context of a circular economy, the plant by-products generated by a company can be transformed from a problem to be solved to a new business opportunity. As a result, this evolution directly impacts the scalability of by-product valorization [150,151].

In the food industry, by-products must not only be nutritionally beneficial but also safe for human consumption [150]. This requires preserving the integrity of bioactive compounds throughout processing, ensuring food safety and optimizing shelf life without compromising product quality. To achieve this, advanced tools like high-performance liquid chromatography (HPLC), mass spectrometry, and nuclear magnetic resonance (NMR) are essential for monitoring the purity and concentration of bioactive compounds throughout the valorization process [152]. Ensuring that these methods are efficiently integrated into production workflows is essential to achieving both technical precision and economic feasibility, particularly as the scale of production increases.

Technological advances supporting the extraction, purification, and commercialization of bioactives from plant by-products are a key factor in making the valorization of plant by-products in biomedicine possible. Over the past five years, there have been significant advancements in the technologies surrounding the commercialization of bioactives from food residues. This includes the development of the advanced extraction techniques mentioned and explained in Table S1, as well as integrated biorefineries. Combining some of these emerging extraction methods (SFE and UAE, for example) can increase the overall efficiency and extract yield, ensuring enough material is available for pharmacological testing. However, these novel extraction protocols and methodologies have limitations and present challenges. Two such limitations are the variability in the composition of plant by-products and the difficulty of scaling up extraction methodologies to high Technology Readiness Levels (TRLs). Once extracted, the stability and activity of bioactive compounds can decrease, so measurements to prevent this are needed. These include optimizing storage conditions, using stabilizing agents, and performing encapsulation (e.g., nanoencapsulation, liposomal encapsulation, spray drying) [153]. This includes the development of advanced extraction techniques and integrated biorefineries. Linked to the advancement of sophisticated extraction techniques and biorefineries, technological progress has led to novel food applications. These applications facilitate the incorporation of bioactive compounds derived from plant by-products into functional foods, nutraceuticals, and food supplements. This development considerably expands their commercial potential. As an example, the combination of green extraction techniques and a biorefinery approach has been shown to be effective in valorizing coffee by-products as bioactive food ingredients and nutraceuticals [154].

Conventional methods of extracting bioactive compounds from plant tissues include Soxhlet extraction, maceration, percolation, and hydro-distillation. Non-conventional, green, or emerging extraction methods include ultrasound-assisted extraction (UAE), microwave-assisted extraction (MAE), enzyme-assisted extraction (EAE), fermentation-assisted extraction (FAE), supercritical fluid extraction (SFE), pressurized liquid extraction (PLE), pulse electric field-assisted extraction (PEFAE), supercritical water extraction (SWE), instant controlled pressure drop (détente instantanée contrôllée, DIC), supercritical liquid chromatography (SFC), pressurized hot water extraction (PHWE), and deep eutectic solvents (DESs). The main advantages of these methods over conventional ones are high-quality extracts, greater selectivity, better isolation, reduced environmental impact, lower energy consumption, and safer products [33]. Each of the advanced extraction techniques mentioned has its own particularities, strengths, and limitations, which are summarized in Table S1.

Biorefineries have the capacity to produce a wide range of pharmaceutical products, including drugs, nutraceuticals, and other health-related products. However, there are some challenges that must be overcome, such as scaling up biological processes for large-scale production, ensuring commercial viability, and adhering to strict regulatory compliance and rigorous quality control to guarantee product safety and efficacy [155]. Technological advances in this area have led to the development of integrated biorefineries. The key distinction between a biorefinery and an integrated biorefinery lies in the extent of product diversification and the technologies employed. While a typical biorefinery focuses on the conversion of biomass into a range of products, an integrated biorefinery uses a broader range of technologies and feedstocks to produce a wider spectrum of valuable products [156]. Indeed, integrated biorefineries have the potential to enable the co-production of biofuels and pharmaceutical compounds through biomass valorization, thus delivering an innovative interdisciplinary solution to the global demands for sustainable energy and advanced therapeutics [155]. Despite this promising perspective, implementing biorefineries to extract and valorize plant by-products for pharmaceutical and cosmetic applications faces several real-world challenges. These include high costs, regulatory hurdles, and low consumer awareness [157,158]. The technological limitations that we have identified are due to the complexity of processing and scaling up and the need to maintain product quality. From an economic perspective, the feasibility of such a venture would depend on various factors, including feedstock costs, product prices, and investment costs. It should be noted that there are inherent uncertainties associated with new technologies and products. Regulatory barriers involve navigating complex regulations and securing necessary permits. Investment challenges are typically associated with the need for substantial upfront costs and the potential for long payback periods. Furthermore, nowadays, that the production of bioproducts with added value for the pharmaceutical industries is efficiently achieved through algae biotechnology applied in biorefineries [157].

A report by the EU in 2018 [159] identified 803 biorefineries within the EU that produced bio-based chemicals, liquid biofuels, composites, and fibers. However, few of them integrated the production of bio-based products (chemicals and/or composites) and bio-based energy (biofuels and/or other types of energy derived from biomass). In most cases, the predominant type of feedstock was agricultural, except in Finland, Sweden, and Portugal, where the most abundant feedstock came from forestry. Ten facilities in France, six in the Netherlands, five in Spain, three in Germany, and three in Ireland employed marine-derived feedstock (including macro/microalgae). More recently, a study commissioned by CEPI (the Confederation of European Paper Industries) in 2024 (https://www.cepi.org/wp-content/uploads/2024/09/Cepi_BioRefineries_Executive-Summary_2024.pdf, accessed on 11 June 2025) revealed the swift growth of the biorefinery sector in Europe. Biorefineries produce environmentally friendly alternatives to materials, chemicals, fuels, energy, food and feed, and pharmaceuticals and cosmetics, which are currently mostly supplied by Europe’s large petrochemical sector.

EU policies support the development of projects focused on smart, integrated energy-driven biorefineries for the co-production of advanced biofuels, biochemicals, and biomaterials (https://ec.europa.eu/info/funding-tenders/opportunities/portal/screen/opportunities/topic-details/horizon-cl5-2024-d3-02-03, accessed on 11 June 2025). One such project is SCALE, developed between 2021 and 2025, which aims to build and operate a flagship plant producing high-nutrient ingredients derived from untapped microalgal diversity for the food, food supplement, feed, and cosmetics sectors through economically sound and environmentally friendly processes. Coordinated by Microphyt (Baillargues, France), the project has been integrated by a wide consortium of participants (research centers and industries) from various countries, including France, Norway, Spain, the United Kingdom, and Belgium.

Moreover, several industrial-scale ventures and patented processes have recently advanced the valorization of plant by-products into bioactives. Arbiom (Paris, France/Durham, NC, USA) operates a continuous fermentation pilot to transform wood and agricultural residues into single-cell protein (SylPro^®^, Arbiom, Paris, France), integrating biomass valorization and regulatory scale-up [160]. Andritz offers its Turbex™ (Höri, Switzerland) extraction system, a commercial low-pressure, green extraction solution that processes fruit peels, and brewers’ bagasse to produce functional food ingredients [161]. Recent patents further highlight industrial innovation in extracting bioactives from plant by-products. WO 2021/119826 A1 [162] describes a method for enriching polyphenols from various plant residues, and patent US 10034910 B2 targets the extraction of flavan-3-ols from Chardonnay grape seed [163].

## 6. Opportunities and Remaining Challenges

The valorization of vegetable residues represents a promising and sustainable strategy that aligns with the principles of the circular economy. In a recent review [164], a list of plant-derived bioactive compounds whose therapeutic application has already been approved is provided. The years of approval range from 1827, when morphine extracted from *Papaver somniferum* was approved for use as an analgesic, to 2021, when samidorphan extracted from the same plant was approved for use in cases of schizophrenia and bipolar disorder. However, despite this promising outlook, there are still many weaknesses in the process of plant by-product recovery in the biomedical field (Table 6).

The first issue of note is the role played by the solvents used to produce the different by-product extracts. The biological effect of plant by-products seems to be closely related to the type of solvent used. The most commonly used solvents are ethanol, methanol, water, or mixtures of these solvents. The mechanism of action underlying this biological effect is therefore solvent-dependent. This means that the observed biological activity is not only a reflection of the inherent properties of the plant but also the result of the specific compounds extracted, which vary depending on the solvent used [169]. Standardization in obtaining plant extracts is therefore a crucial aspect to ensure consistent replication of the effects observed in vitro or in vivo in a subsequent medical application. Such standardization, however, needs much improvement to date. The application of rigorous quality control procedures, supported by precise legislation, is key to ensuring the safety and efficacy of products obtained from natural sources [170]. Another essential aspect is to have a detailed profile of the extracted compounds using different solvents, as well as their concentration and the presence of possible impurities. This will ensure a better understanding of the biological effects, allow more reliable and reproducible studies, and enhance the safety and effectiveness of the plant compounds obtained. The application of modern techniques such as high-throughput screening and computational tools can make it possible to test a massive number of extracts for compounds with therapeutic potential in a short time [170]. On the other hand, it is highly desirable that the production of plant extracts is carried out in the most environmentally sustainable way possible, which supports the importance of research into emerging ‘green’ extraction technologies [171].

Research aimed at discovering the biochemical and molecular mechanisms underlying the action of active compounds extracted from plants has mostly been carried out using compounds isolated individually from individual plants. However, it has been shown that the therapeutic activity of isolated compounds may be lower than that of the extract from which they were obtained [172]. This is due to possible synergistic effects between several of the compounds included in the extract. Possible interactions between different compounds present in the same extract and synergistic, additive, or antagonistic effects have been detected between bioactive compounds extracted from plants and medicines, with the consequence that the pharmacokinetics and pharmacodynamics of medicines may be altered [153,165]. For instance, mandarin and lemon peels are rich in flavonoids such as hesperidin and naringenin (Table 2 and Table 4), which can inhibit cytochrome P450 enzymes (CYP3A4), potentially increasing the bioavailability of drugs like statins or certain chemotherapeutics [173]. Banana peels (Table 1) are a source of catecholamines like dopamine, which could interact with medications affecting the neurotransmitter balance, such as antidepressants [174]. Enzyme assays can study enzymes like CYP450 to understand compound metabolism and identify potential interactions [167]. However, not all interactions are detrimental; some can even be beneficial. For example, grape seed extract (Table 4) has shown synergy with chemotherapy drugs, enhancing their anticancer effects and potentially reducing the toxicity of treatments like cisplatin.

To date, fewer natural products have entered clinical trials than synthetic compounds. However, tested bioactive products tend to show more favorable results in early clinical phases, mainly due to lower toxicity profiles than synthetic compounds [42]. A very relevant aspect to obtain crucial information on the behavior of plant bioactive compounds in the body is the study of ADME properties. After consumption, the absorption of phytochemicals can be influenced by the pH of the gastrointestinal tract, their distribution and ability to reach target sites can be modified by their affinity for plasma proteins, and gut microbiota or phase II metabolism can transform these compounds, potentially inactivating them or altering their activity. Fernandez-Ochoa et al. [167] concluded that in vitro assays will provide a comprehensive understanding of how bioactive compounds from a plant by-product extract behave in the digestive system, supporting the transition to in vivo studies and clinical trials.

The importance of standardizing the extraction of plant extracts has been discussed above, but this is not the only aspect in need of standardization. Storage conditions at waste production sites, transport to the processing site, transit time, and handling practices can lead to degradation or loss of bioactive compounds. In other words, transport and logistics need to be supported by standardized protocols to ensure the consistency and efficacy of the material [5]. Fortunately, cold-chain monitoring systems and wireless Internet of Things (IoT) technologies, such as radiofrequency identification (RFID), allow for tracking the movement of plant by-products in real time, providing valuable data on environmental conditions (temperature, humidity, etc.) during transit [175,176].

Throughout this presentation, we have made clear the great importance of standardizing processes and protocols in order to convert plant by-products into a source of bioactive compounds with biomedical potential. However, there is a key factor that is difficult to standardize: the composition and quality of the residues generated. Even in cases where certain medicinal plants are used to extract bioactive compounds, there are aspects that are difficult to control, such as the age of the plant at the time of harvesting (very difficult to know in perennial plants) and the growing conditions (highly variable in plants from the field, where they have been exposed to a changing climate and have grown on different soils with different nutritional inputs, for example). The species or variety within a plant genus, the organ where the active compound is synthesized or stored, and the specific time in the plant’s life cycle are also aspects that lead to variability in the composition and levels of bioactive compounds [164]. Along the agri-food chain, these aspects may be fairly well known at the level of the primary sector that grows the plants (farmers), less well known in industrial processes that work with the raw material provided by the primary sector (juice companies, for example, who may use the same trusted suppliers), and completely unknown at the household and catering levels. In the latter areas, moreover, organic residue recycling does not currently discriminate between animal and vegetable residues, nor is it prepared for the specific recycling of certain vegetable residues of interest due to the properties of the bioactive compounds they can store. Even if animal residues are separated from vegetable ones, plant by-products will consist of random mixtures of different plants, which will vary throughout the year depending on the seasonal products. In addition, in the domestic environment, the mixture of plant by-products includes raw material and material that has undergone different cooking techniques (boiling, frying, etc.) that can modify the properties of the active compounds. In addition, the conditions of temporary storage of household waste (time, temperature, humidity), both at home and in urban containers, can also alter these properties and accelerate the microbial spoilage of by-products [150]. Current household waste collection systems must be restructured to enable the reuse of plant-based residues for bio-health applications. This requires staff training in the hospitality sector and early civic education to foster proper recycling habits, aligning with the environmental education goals of the European Union (European Higher Education Area) [177]. There are concrete examples of the efforts made in citizens’ education. The EU-funded SchoolFood4Change project (https://schoolfood4change.eu/, accessed on 12 June 2025) develops innovative solutions and locally adaptable good practices for schools, ultimately aiming to achieve the ambitious goal of enabling community-wide food system change. Moreover, **t**he Agro2Circular project (https://agro2circular.eu, accessed on 12 June 2025), coordinated by CETEC in Spain and comprising several universities, industries, and financial entities, encourages citizens to participate in the regular and selective collection of agri-food waste. This waste is then transported to the National Technological Centre for Canning, where its active ingredients are extracted for use in nutritional formulas, cosmetics, food, and other high-value products. The project aims to promote acceptance of the circular economy in local communities by raising awareness and providing information to citizens, as well as collecting waste.

Once these residues have been collected, the application of Food Industry 4.0 technologies, such as Artificial Intelligence, data science, bioinformatics, smart sensors, robotics, or digital twins would allow the transformation of plant residues into revalued products [168]. We propose that these technologies, if their cost-effectiveness and scalability are positive, should be used to carry out an initial screening to check their quality and, based on the results, to decide on their final use. VCG.AI (https://vcg.ai/, accessed on 11 June 2025) is a company that uses intelligent algorithms to match by-products with proven, value-adding technologies and high-demand markets. It delivers solutions that maximize economic and environmental benefits. The company developed the ‘Data-driven food waste valorization for a large food retail chain’ project to extract lycopene and pectin from fruit and vegetable waste in order to manufacture pigments, dyes, cocoa, sugar confectionery, and non-distilled fermented beverages.

Poorer-quality batches of residues could be diverted to composting for application as fertilizer, for example. However, today, this proposition is more feasible in developed countries, which possess state-of-the-art machinery and processes that can transform by-products into valuable products [178], as well as strong regulatory frameworks and financial incentives [179]. In contrast, developing countries often face significant challenges in the area of by-product recycling due to their limited access to advanced technologies. Nowadays, there are numerous documents focused on the regulation of herbal medicinal products, but the legislation dealing with plant-based by-products is very scarce. Moreover, the regulation of the utilization of plant by-products in biomedicine or nutrition can also vary across countries [170].

Despite the significant limitations that remain to be overcome, the use of plant by-products, whether in biomedicine or nutrition, presents significant potential for advancing both fields. Not only are these by-products cheap and highly available, but they also offer an invaluable and sustainable resource for developing new therapeutic solutions to combat devastating diseases such as those discussed in this article [180]. Favorable laws and the availability of both public and private funding encourage their application, particularly through investments in research and scalability. The growing market for these resources could generate new employment opportunities and companies could also benefit by selling their plant by-products to third parties, further expanding the value chain.

## 7. Conclusions

The valorization of vegetable residues represents a promising and sustainable strategy that aligns with the principles of the circular economy, turning large amounts of agro-industrial by-products into valuable raw materials for diverse applications in fields such as biomedicine, pharmaceuticals, nutrition, and dermocosmetics.

Despite ongoing weaknesses and challenges, such as the lack of standardized extraction and processing methods, the field holds considerable strengths and opportunities. Advances in green extraction methods have significantly improved the efficiency and quality of bioactive compounds obtained from these residues, and the development and implementation of several biorefineries demonstrate the practical feasibility of these technologies. Moreover, consumer acceptance is gradually increasing, driven by stricter safety regulations, growing awareness of sustainability, educational programs promoting best practices, and an expanding body of scientific evidence supporting the benefits of plant-based bioactives.

These combined efforts pave the way for a more sustainable and innovative future, unlocking the full potential of vegetable residues across multiple industries. This not only enhances resource efficiency and promotes zero-waste goals but also offers a pathway to more resilient and health-conscious production systems in the context of circular bioeconomy models.

## Figures and Tables

**Table 1:** Antibacterial effects of plant residues.

Plant	By-Product	Bioactive Compounds	Type of Extractant	Bacterial Species	Activity (MIC: mg By-Product mL^−1^ Extract)	Reference
Pomegranate (*Punica granatum*)	Peel	Polyphenols, carotenoids, and tannins	Methanol 70%	*E. coli (CCM 3988)* *S. entérica (CCM 3807)* *E. faecalis (CCM 4224)* *S. aureus (CCM 2461)* *B. subtilis (CCM 1999)*	PTO8 HIC 17.1 255 93 21.3 51.6 12.5 68.5 57.2 1.5 3.4	[23]
Orange (*Citrus* spp.)	Peel	Not specified	Methanol	*K. pneumoniae* *E. coli* *Salmonella typhi* *Streptococcus pyogenes*	MIC: μg mL^−1^ 310 320 280 370	[50]
			Distilled water	*K. pneumoniae* *E. coli* *S. typhi* *S. pyogenes*	140 270 250 340	
Yellow lemon (*Citrus medica limonum*)	Peel		Methanol	*K. pneumoniae* *E. coli* *S. typhi* *S. pyogenes*	130 220 210 210	
			Distilled water	*K. pneumoniae* *E. coli* *S. typhi* *S. pyogenes*	310 140 280 250	
Ginger (*Zingiber* *officinale*)	Peel	Polyphenol	Methanol	*E. coli* *Bacillus megaterium* *B. cereus* *S. aureus*	Not determined	[47]
Garlic (*Allium sativum*)						
Onion (*Allium cepa*)						
Potato (*Solanum tuberosum*)						
Tomato (*Solanum lycopersicum*)	Peel	Lycopene, β-carotene, lutein, and different phenolic compounds	Methanol 80%	*S. aureus (ATCC 49444)* *B. subtilis (ATCC 11778)* *L. monocytogenes (ATCC 19114)* *K. pneumoniae (DSMZ 2026)*	Tiny Tim 0.625 1.25 2.5 2.5	[49]
Banana (*Musa acuminata*)	Peel	Flavonoids, quinnones, and alkaloid	Ethanol/distilled water	*P. aeruginosa* *E. coli* *B. subtilis*	3.125/3.125 3.125/3.125 3.125/3.125	[51]
	Pulp	Not specified	Methanol	*S. aureus* *Streptococcus pneunoniae* *E. coli* *Haemophilus influenza*	Not determined	[47]
	Leaves	Alkaloids, saponins, terpenoids, tannins, phenols, and flavonoids	Methanol and hexane	*Staphylococcus* *epidermidis*	Not determined	[48]
Black cumin (*Nigella sativa*)	Seed oil	*p*-cymene, linalool, thymoquinone, *trans*-anethole, and *m*-thymol	Not specified	MRSA *^#^*	0.0001	[54]

^#^ Six non-replicate clinical MRSA isolated from specimens of blood, sputum, pus, and wound swabs were used for the investigation. MRSA turned out to be resistant to the following antibiotics: augmentin, benzyl penicillin, oxacillin, cefuroxime, cefuroxime + axetil, imipenem, ciprofloxacin, levofloxacin, sulfamethoxazole + trimethoprim, tetracycline, and rifampicin.

**Table 2:** Antifungal effects of plant residues.

Plant	Organ	Vegetal Residue	Fungal Species	Type of Extractant	Activity (Growth Inhibition)	Disease	References
Pomegranate (*P. granatum*)	Fruit	Peel	*Aspergillus flavus*	Methanol 70%	* ••	Pulmonar aspergillosis Hepatocellular carcinoma (aflatoxin B1, B2, M1, M2)	[23]
			*Aspergillus parasiticus*		•	Aspergillosis (aflatoxin)	
			*Fusarium verticillioides*		•••	Keratitis	
Ginger (*Z. officinale*)	Rhizome	Peel	*F. verticillioides*	Methanol	•••	Keratitis	[47]
Garlic (*A.* *sativum*)	Bulb	Peel			•••		
Onion (*A. cepa*)	Bulb	Peel			•		
Potato (*S. tuberosum*)	Bulb	Peel			•••		
Banana (*M. acuminata*)	Leaf	Leaf	*Trichophyton mentagrophytes*	Hexane and methanol	•••	Ringworm	[48]
Banana (*M. paradisiaca*)	Fruit	Peel	*A. flavus*	Distilled water	••	Pulmonar aspergillosis Hepatocellular carcinoma (aflatoxin B1, B2, M1, M2)	[65,66]
			*Penicillium griseofulvum*		••	Alimentary intoxication (patulin)	
			*A. tubingensis*		•	Nefrotoxicity (ocratoxin A)	
Mandarin (*C. reticulata*)	Fruit	Peel	*A. flavus*		••	Pulmonar aspergillosis Hepatocellular carcinoma (aflatoxin B1, B2, M1, M2)	
			*A. tubingensis*		•	Nefrotoxicity (ocratoxin A)	
			*P. griseofulvum*		•••	Alimentary intoxication (patulin)	

* Growth inhibition achieved with the *n*-butanolic fraction. • Low activity. •• Medium activity. ••• High activity.

**Table 4:** Antitumoral effects of plant residues.

Vegetal Residue		Bioactive Compounds	Type of Extractant	In Vitro Activity as Antitumorals	Reference
Mango (*Mangifera indica*)	Mango seed kernels	Gallate derivatives and gallotannins, mangiferin	Lipidic fraction: *n*-heptane Polar fraction: ethanol/ethyl acetate	Colorectal cancer (HT-29)	[93]
Orange, lemon, mandarin… (*Citrus* spp.)	Citrus peel oils	Limonene, hesperetin	Water	*C. reticulata*: Dalton’s Lymphoma Ascites (DLA) cells	[94]
Grape (*Vitis vinifera*)	Grape leaves	Polyphenols	98% (*v*/*v*) methanol	Breast, leukemia, and lung	[95]
	Grape seeds	Flavan-3-ols	Ethanol/water solution (50:50 *v*/*v*)	Colorectal cancer (Caco-2)	[96]
		Proanthocyanidin (GSPE)	Water-alcohol	GSPE may be a promising adjuvant to prevent cardiotoxicity of doxorubicin	[97]
		Procyanidin B2	Not specified	Colorectal cancer	[98]
Pomegranate (*P. granatum*, Akko variety)	Pericarp (peel powder)	β-glucans	Acetone and methanol	Chemopreventive and adjuvant treatment	[99,100]
Pink lady apple (*Malus domestica*)	Peel	Flavonoids	Ethyl alcohol	LoVo human colon cancer cells and MCF-7 human breast cancer cells	[101]
Sweet potato (*Ipomoea batatas*)	Peel	Nanoemulsion of carotenoids	Hexane/ethanol/ acetone/toluene (10:6:7:7, *v*/*v*/*v*/*v*)	MCF-7 breast cancer	[102]
Avocado (*Persea americana*)	Seed	Triterpenoids isolates	Ethanol	Liver cancer	[103]
Açaí (*Euterpe oleracea*)	Seed	Phenols	Ethanol + water	A549 cell line (lung cancer)	[104]
		Catechins, procyanidins, phenols	Water	NCI-H460 cell line (lung cancer)	[105]
Camu-camu (*Myrciaria dubia*)	Seed	Phenols, condensed tannins, non-tannin phenolics	Ethanol + water	A549 cell line (lung cancer)	[106]
		Phenols, condensed tannins	Water + ethanol + propanone (41:16:43)		[107]

**Table 5:** Vegetal residues with promising properties as prebiotics.

Vegetal Residue	By-Product	Type of Extractant	Activity	Reference
Yellow watermelon (*Citrullus lanatus*), honeydew (*Cucumis melo*), and papaya (*Carica papaya* Linn.)	Peel	Ethanol and distilled water	Prebiotics	[138]
Orange (*C. sinensis*)	Peel	Not specified	Prebiotics	[136]
Olive (*O. europaea*)	JAO			
Apple (*M. domestica* ‘Gala’), banana (*M. acuminata* Cavendish Subgroup), and passion fruit (*Passiflora edulis*)	Peel	Not specified	Prebiotics	[137]

**Table 6:** SWOT analysis of the situation.

Internal Analysis	External Analysis
**Weaknesses**	**Threats**
There are significant limitations in the current system for recycling domestic and catering organic residues: (i) high variability in the composition; (ii) mixture of animal and plant by-products; (ii) impossibility of obtaining homogeneous raw material for the extraction of bioactive compounds; (iv) lack of protocols for the storage of by-products at home and in the urban core. Short half-life of plant by-products due to microbial spoilage. Solvent-dependent bioactivity. Lack of standardized preparation protocols. Variability in bioactive compound profiles. Limited knowledge of ADME properties. Need for more detailed in vitro analysis [116]. Limited knowledge of interactions between different bioactive compounds and between bioactive compounds and drugs. Low–medium level of education of citizens on waste recycling.	Scarce clinical trials. Variability in raw material composition. Stability issues during transport and storage. Competition from synthetic alternatives. Regulatory and scalability challenges. Different rules across countries [165]. Undefined appropriate valorization approaches to reduce by-product management costs and conserve resources. Little collaboration between manufacturing industry, governments, public, and scientists to implement by-product management.
**Strengths**	**Opportunities**
Sustainability and low environmental impact [166]. Low cost and high availability of plant residues. Diverse applications in medicine, dermocosmetics, and nutraceuticals [27,31,32]. Favorable toxicity profile compared to synthetic alternatives. Growing body of scientific evidence supporting efficacy [167].	Increasing demand for sustainable and natural products. Advances in green extraction technologies [166]. Expanding nutraceutical and functional food markets. Availability of public and private funding for research [34]. Potential for creating new value chains and job opportunities. Economic benefits for waste-producing companies by repurposing or selling by-products. Development of resistance to common antimicrobials: need for research into new molecules. Modernization of the agro-food sector through the application of 4.0 technologies [168]. Increased competitiveness of agro-food companies that adopt circular economy principles and digital innovation [168]. Reduction in the amount of medicinal natural flora collected for extraction of active compounds.

## Data Availability

No data was used for the research described in the article.

## Supplementary Materials

The following supporting information can be downloaded at https://www.mdpi.com/article/10.3390/antiox14080942/s1, Table S1: Strengths, limitations and applications of the main innovative-green-emerging extraction methods. Examples of bioactive compounds extracted from plant by-products applying the mentioned emerging methods. References [181,182,183,184,185,186,187,188,189,190,191,192,193,194,195,196,197,198] are cited in the supplementary materials.
