# Supplementary material for: Potential Application of Plant By-Products in Biomedicine: From Current Knowledge to Future Opportunities
Source: Antioxidants (Basel). 2025 Jul 31;14(8):942. doi: 10.3390/antiox14080942 (PMC12382630; doi:10.3390/antiox14080942)
Supplement: Supplementary file 1 [file antioxidants-14-00942-s001.zip › antioxidants-3671559-supplementary.pdf]

Table S1. Strengths, limitations and applications of the main innovative-green-emerging extraction methods [181–186]. Examples of bioactive compounds extracted from plant by-products applying the mentioned emerging methods.

Method	Description	Advantages/strengths	Disadvantages/limitations	Applications	Examples Bioactive compounds/plant by products
**UAE**	High-frequency sound waves to enhance the extraction of compounds from solid materials (non-thermal process)	● Faster extraction<br>● Reduced solvent use<br>● Improved extraction yields<br>● Increased bioavailability<br>● Versatility<br>● Preserves heat-sensitive compounds<br>● Cost-Effective<br>● Green Technology<br>● Scalability	● Expensive equipment<br>● Energy efficiency<br>● Potential for degradation (higher ultrasonic intensity or duration may lead to degradation of heat-sensitive compounds)<br>● Scalability challenges due to varying energy inputs<br>● Difficulties for optimizing parameters (time, temperature, solvent, power)	● Extraction of bioactive compounds from plants and algae<br>● Extraction of bioactive compounds from fruit and vegetable processing by-products<br>● Extraction of plant natural products to develop new pharmaceuticals or cosmetic ingredients	Antioxidants/Orange peels<br>[187]
**MAE**	Microwave energy to heat solvents in contact with a sample in order to partition analytes from the sample	● High speed<br>● High efficiency<br>● High yield and purity<br>● Reduced solvent use	● Reduced efficiency with non-polar or volatile compounds<br>● Inhomogeneous heating<br>● Need for additional processing (sometimes filtration or	● Extraction of bioactive compounds from plants and food samples<br>● Synthesis and analysis of pharmaceutical products	Polyphenols/Pineapple peel<br>[188]

	matrix into the solvent	● More cost-effective (reduced solvent consumption, shorter processing time, potentially lower equipment costs)<br>● Ease use<br>● Suitable for thermolabile compounds (rapid heating)	centrifugation to remove solid residues)<br>● Possible lipid burning<br>● Availability of equipment (relatively inexpensive, but with limited availability and accessibility in certain settings)	● Environmental analysis. Extraction of pollutants from environmental samples<br>● Other applications in animal tissue analysis, pulp and fiber extraction	
**SFE**	Supercritical fluid to extract a material	● High extraction yields in a short time<br>● Minimal solvent usage<br>● Preservation of thermally labile compounds<br>● Tunable solvent density<br>● Reduced energy consumption<br>● No solvent residue	● High capital cost<br>● High pressure requirements<br>● Complexity of recycling<br>● Limited polar compound extraction<br>● Technical expertise required<br>● Potential for non-reproducibility<br>● May not be suitable for all samples<br>● Moisture requirements (raw material may need freeze-drying)	● Food Industry. Extraction of natural products, bioactive compounds, fats, oils.<br>● Pharmaceutical industry. Extraction of active pharmaceutical ingredients<br>● Other industries. Fractionation of polymeric materials, extraction of environmental pollutants	Polyphenols, anthocyanins/Apple peels<br>[189]
**PLE**	Elevated temperature and pressure to extract compounds from	● Fast extraction<br>● Reduced solvent consumption	● High equipment cost<br>● Optimization required for each application	● Extraction of bioactive compounds from plants and pharmaceutical samples	Antioxidants/ Pomegranate peel

	solid or semi-solid samples	● Automation (multiple samples with minimal human intervention)<br>● Improved selectivity (adjusting temperature and pressure, the polarity of the solvent can be altered and therefore enhances the extraction of specific compounds)<br>● Enhanced recovery of sensitive compounds that may be degraded during conventional extraction<br>● Versatility	● Potential degradation of thermolabile compounds<br>● High-moisture samples may require drying before extraction to avoid issues with co-extracted water<br>● Limited applicability for extracting very polar or ionic compounds<br>● Waste management (extract may require evaporation thus leading to waste generation)	● Food and beverage industry.<br>Extraction of nutrients and natural products, contaminant removal, food waste valorization<br>● Chemical manufacturing processes for extraction or purification.<br>● Environmental monitoring of pollution levels (including emerging contaminants like nitrosamines, alkyl phenols, bisphenol A, and UV filters). Extraction of contaminants (pesticides, heavy metals) from various environmental matrices	[190]
**PEFAE**	High-voltage electric pulses to enhance the extraction of bioactive compounds from biological matrices, including food waste	● Improved extraction efficiency (increased extraction yield, reduced solvents and energy)<br>● Reduced degradation of heat-sensitive compounds (due to reduced extraction time and temperature)<br>● Selective extraction of specific compounds	● High initial investment<br>● Effectiveness dependent on conductivity (high conductivity can affect the electroporation process)<br>● Temperature management<br>● Need for optimization for different materials<br>● Potential for side reactions (At high intensities, PEF can lead to	● Extraction of bioactive compounds, antioxidants, vitamins, pigments from food waste, plant tissues and microbial cells. Food waste valorization.<br>● Extraction of valuable compounds (proteins, polysaccharides, lipids, and pigments) from algal biomass.	Hesperidin/Tangerine peel<br>[191]

		● High quality of the extracted compounds (non-thermal processing)<br>● Potential for Industrial Applications	changes in the extracted compounds)<br>● Electrochemical reactions may impact the food product, produce electrode corrosion, fouling and electrolysis of water	● Food processing to improve the quality and shelf life of food products.<br>● Biotechnology and medicine (cell manipulation and gene therapy)<br>● Enzymatic extraction enhancement<br>● Others: extraction of minerals	
**EAE**	Enzymes to enhance the release of bioactive compounds from plant and other biological sources	● High specificity<br>● High efficiency and reproducibility<br>● Reduced extraction time<br>● Lower solvent consumption<br>● Improved extraction efficiency<br>● High quality extracts, with fewer impurities<br>● Cost-Effective. Initial cost of enzymes can be high, but the overall process can be more cost-effective due to reduced solvent consumption and time	● High enzyme costs of enzymes for large-scale applications<br>● Environmental conditions. Temperature, pH, and other environmental factors affect enzyme activity<br>● Limited enzyme availability. Finding the right enzyme can be challenging.<br>● Limited enzyme stability under industrial conditions<br>● Potential for degradation of compounds under certain conditions	● Waste and plant by-products utilization for the extraction of valuable and bioactive compounds with potential therapeutic properties for the pharmaceutical and nutraceutical sectors<br>● Valorization of plant by-products for enzyme production, sugar and fiber extraction<br>● Food and Beverage industry. Improved quality of products and enhanced functional properties	Anthocyanins/Eggplant peel<br>[192]

		● Enzymes work under mild conditions, preserving the integrity of extracted compounds<br>● Enzymes can increase the solubility of certain compounds making them easier to extract	● Safety concerns. Some enzymes can cause allergic reactions or skin irritation<br>● Scalability challenges. Some enzymes may be less effective or more expensive at larger scales		
**FAE**	Fermentation to increase the extraction of bioactive compounds from plant materials	● Economically viable<br>● Improved extraction<br>● Milder extraction conditions minimize the risk of thermal degradation of sensitive compounds<br>● High yield<br>● Reduced solvent use	● Slow process<br>● Lower overall extraction rates in certain situations<br>● Fermentation optimization can be complex<br>● When fermentation processes are batch processes, they become labor-intensive and potentially less scalable<br>● Difficulties for maintaining optima microbial growth in large-scale bioreactors<br>● Dependent on the readily available carbon sources for starting the fermentation and for	● Pharmaceutical industry. Extracts may possess antibacterial, antifungal or anti-inflammatory effects<br>● Food and beverage industry. Production of yogurt, cheese, sauerkraut and alcoholic beverages. Possibility of enhancing the nutritional value and functional properties of these products<br>● Chemical industry. Production of ethanol from various sources<br>● Agricultural Biotechnology. **I**mproved crop yields and enhanced nutritional value of agricultural products	Ellagic acid/ Mexican rambutan peel<br>[193]

			the sustained growth of fermenting microorganisms<br>● Possible presence of unwanted microorganisms which reduce yield and final product quality	● Environmental Technology. Biological wastewater treatment	
**SWE**	Uses water in its supercritical state (373°C and 220 bars) as a solvent	● Rapid extraction<br>● Increased selective extraction of specific compounds<br>● Reduced solvent and energy used<br>● Environmental friendliness. Supercritical fluids can be non-toxic, non-flammable, and leave no harmful residues<br>● Preservation of labile compounds<br>● Tunable solvent density to optimize extraction	● High equipment and operational costs<br>● Technical complexity<br>● Limitations for extracting highly polar compounds<br>● Cosolvent requirement for certain extractions (thus increasing complexity and cost)<br>● Freeze-drying of raw material for moisture reduction	● Waste management and recycling. Extraction of natural products and lipids from plant materials and other sources<br>● Pharmaceutical industry. Extraction of bioactive compounds from natural sources for drug development<br>● Food and beverage industry<br>● Biotechnology and biomedical engineering. Extraction, purification and formulation of bioactive compounds and biomaterials<br>● Chemical industry. Catalysis, polymerization, and other chemical processes<br>● Energy sector. Extraction of hydrocarbons from natural sources and processing biofuels	Phenolic compounds/Potato peel<br>[194]

				● Materials science. Processing polymers, creating nanoparticles, and developing new materials	
**DIC**	Thermomechanical process where a material is rapidly exposed to high-pressure saturated steam followed by a quick reduction in pressure towards a vacuum	● Enhanced extraction<br>● Reduced processing time<br>● Improved product quality. Preservation of nutritive value and functional behavior<br>● Energy efficiency<br>● Decontamination of microorganisms (vegetative and spore forms)<br>● Green technology (efficient use of water vapor and reduced solvent consumption)<br>● Versatility. Can be applied to a wide range of materials	● Scalability challenges from laboratory to industrial levels.<br>● Cost considerations<br>● Potential sensory changes<br>● Specific limited applications. Not suitable for all types of materials or applications. Optimization may require specific products	● Food processing (drying, texturing, decontamination, reduced anti-nutritional factors)<br>● Extraction of bioactive compounds (essential oils, phenolics, flavonoids) from various food sources.<br>● Pharmaceutical industry. Pharmaceutical processing and decontamination.<br>● Others: Animal feed processing. Decontamination and preservation of fresh cut fruits and vegetables.	Phenolic compouds/ Pomegranate peel<br>[195]
**SFC**	Uses a supercritical fluid, like carbon dioxide (CO_2), as the mobile phase	● Fast analysis and high resolution<br>● Environmental friendliness<br>● Minimal sample preparation	● Complexity. Specialized high-pressure equipment required and careful temperature control<br>● Not adequate for highly polar compounds	● Analysis of natural products and plant extracts. Very useful for thermally sensitive compounds<br>● Pharmaceuticals. Drug development and quality control (separation and analysis of drug formulations, chiral separations,	Polymethoxyflavones/Orange peel

		● Cleanliness. Minimal contamination from the mobile phase.<br>● Compatibility with a wide range of samples<br>● Sensitivity and reduced matrix interference	● The large volumes of modern SFC systems can affect the use of certain column sizes<br>● Potential for isobaric interferences due to short run times	impurity detection and lipophilicity determination)<br>● Food and beverages. Analysis of food contaminants, flavor compounds, and the composition of food products.<br>● Environmental analysis of pollutants and contaminants in water, soil, and air<br>● Others: cosmetic product analysis, petrochemical industry, polymer analysis, pesticide analysis, surfactant analysis	[196]
**PHWE**	Uses hot water under pressure to extract bioactive compounds from various matrices	● Environmental friendliness because it uses water as a solvent<br>● Fast extraction<br>● Enhanced solubility due to pressure and heat conditions<br>● High extraction yields<br>● Versatile to extract a broad range of bioactive compounds and secondary metabolites	● Potential degradation of thermally labile compounds<br>● Unwanted reactions (hydrolysis, Maillard reactions, caramelization) leading to undesirable products and erroneous results<br>● Optimization required to find the optimal conditions (temperature, pressure, time)	● Extraction of natural products<br>● Food Industry. Extraction of plant bioactive compounds as food additives, nutraceuticals, and ingredients in processed foods.<br>● Pharmaceutical industry. Extraction of plant bioactive compounds with anti-viral, anti-inflammatory, and antioxidant properties<br>● Environmental monitoring. Extraction of organic contaminants	Phenolics (eriocitrin and hesperidin)/Lemon peel

		● Reduced cost for its simplicity and the use of water as a solvent<br>● Can be adapted for both laboratory-scale and industrial-scale applications	● Extraction of non-target compounds and thus the need of further purification steps.<br>● Complexity. Specialized equipment and expertise required, especially for large-scale applications.	from foodstuff and soil/sediments for analysis<br>● Biorefinery. Extraction of hemicellulose and others from wood<br>● Extraction of biopolymers from algae	[197]
**DES**	Mixtures of two or more solid compounds that form a liquid phase at room temperature	● Environmental friendliness. Most solvents have low toxicity and are biodegradable<br>● Low cost<br>● Ease preparation<br>● Versatile properties	● Potential toxicity in some cases. Incomplete understanding of toxicity.<br>● High viscosity<br>● Limited applications when highly polar or highly volatile solvents are required<br>● Absorption of water, which can affect properties and applications<br>● Challenges in recycling in some cases to avoid environmental contamination	● Food Science. Extraction of bioactive compounds removing heavy metals.<br>● Chemistry:<br>◦ Green Chemistry<br>◦ Chemical Reactions<br>◦ Analytical Chemistry<br>● Biotechnology and bioanalysis. Biotransformations, modification of biopolymers (cellulose, starch), drug delivery<br>● Metal Processing (electroplating, electro-polishing and metal extraction)	Phenolics/ Orange peel<br>[198]

		• Nanotechnology. Synthesis of nanomaterials. Development of new materials for energy storage<br>• Sensors and biosensors
